# Panel-Aware Local Background Filtering for Photovoltaic Thermal Anomaly Detection and Automatic Bounding-Box Pre-Annotation

**DOI:** 10.3390/s26196163

**Published:** 2026-09-29

**Authors:** Gulhan Ustabas Kaya, Esra Aga, Duygu Demircan, Hakan Kaya

**Affiliations:** Department of Electrical-Electronics Engineering, Zongudak Bulent Ecevit University, 67100 Zonguldak, Türkiye; esra.a@fbe.karaelmas.edu.tr (E.A.); duygu.demircan@beun.edu.tr (D.D.); hakan.kaya@beun.edu.tr (H.K.)

**Keywords:** photovoltaic systems, hotspot, local background filtering, fault detection, automated annotation

## Abstract

Thermal imaging is widely used for identifying abnormal thermal patterns in photovoltaic (PV) systems. However, complex backgrounds, varying thermal conditions, and environmental factors can reduce the spatial reliability of thermal-anomaly localization, particularly under real operating conditions. This study proposes a training-free image-processing framework that integrates panel-centered analysis, inner-panel masking, panel-coverage control, and local-background filtering to detect thermal-anomaly candidates and automatically generate bounding-box pre-annotations for subsequent deep learning applications. The framework was evaluated using five images from a publicly available thermal PV dataset and four independently acquired UAV-based field images from a grid-connected rooftop PV system at Zonguldak Bülent Ecevit University (BEUN). Compared with global thresholding and a panel-constrained Otsu-based baseline, the proposed method generally reduced redundant detections and reduced the absolute number of out-of-region detections while retaining regions showing spatial agreement with the image-derived pseudo-reference. Evaluation on the pseudo-color field images demonstrated promising applicability under real operating conditions. Moreover, a clear domain shift and increased out-of-region detections in some images indicate that further improvement in robustness is required for pseudo-color thermal representations and complex real-world conditions. Therefore, the present results are regarded as preliminary proof-of-concept evidence rather than broad validation of robustness or field-deployment readiness. The framework is computationally lightweight and shows potential for near-real-time processing.

## 1. Introduction

Thermal imaging is a widely adopted technique for fault diagnosis in solar photovoltaic (PV) systems. However, its effectiveness is often degraded by low luminance conditions caused by radiative heat loss, object-to-sensor distance, and unfavorable reflection angles. These factors reduce thermal contrast and impair vision-based analysis, while conventional contrast enhancement techniques frequently introduce noise amplification, background artifacts, and brightness distortion, further limiting diagnostic reliability [1]. Among PV faults, hotspot anomalies, which are characterized by localized excessive heating, pose a critical threat to system efficiency, operational safety, and long-term reliability, therefore making their accurate and timely detection essential to minimize energy losses and lifecycle costs [2,3].

Existing hotspot detection approaches based on handcrafted features extraction, traditional machine learning, and deep learning exhibit notable limitations in real-world PV environments. Traditional machine learning methods suffer from limited generalization due to high-dimensional, dataset-specific feature representations that are computationally expensive to extract and poorly transferable across different PV technologies and climatic conditions. Although convolutional neural networks (CNNs) achieve high diagnostic accuracy by learning complex thermal patterns, they require large annotated datasets, substantial computational resources, and careful hyperparameter tuning. Moreover, they remain vulnerable to overfitting under varying environmental and installation conditions [2,3].

### 1.1. Related Work

To overcome these challenges, a wide range of image-processing and learning-based algorithms have been developed for PV hotspot detection and segmentation, where accurate isolation of defective regions plays a central role. Early studies relied on region of interest (ROI) analysis, contour extraction, histogram-based features, classical edge detection, and morphological operations to localize defective cells and shading-induced hotspots, yielding promising results under controlled conditions [4,5,6]. Subsequent studies combined conventional image preprocessing, including Gaussian filtering, Laplacian-based edge enhancement, and threshold-based segmentation, with CNNs to improve the automation and robustness of hotspot identification [7].

With the advancement of deep learning, CNN-based architectures have become increasingly prominent in PV fault analysis. Encoder–decoder architectures such as U-shaped Convolutional Neural Network (U-Net) and Semantic Segmentation Network (SegNet), instance segmentation frameworks such as Mask Region-Based Convolutional Neural Network (R-CNN), and object-detection approaches based on You Only Look Once (YOLO) have been investigated for PV fault detection and hotspot localization, including applications involving UAV-based thermographic inspection [8,9,10]. More recently, multi-stage approaches have been introduced to reduce interference from complex backgrounds and improve hotspot localization. In particular, the Two-Tier Semantic Segmentation (2TSS) framework first separates PV modules from the background and subsequently performs refined hotspot segmentation. Comparative experiments with U-Shaped Convolutional Neural Network (U-Net), Residual Neural Network (ResNet-18), and ResNet-50 demonstrated the effectiveness of this hierarchical strategy, while image enhancement prior to model training provided an approximately 2.26% improvement in hotspot segmentation accuracy [11]. A recent multiclass U-Net approach has similarly demonstrated the feasibility of jointly distinguishing the PV panel, hotspot, and background within thermal imagery [12]. These studies indicate that explicitly separating the panel region from irrelevant background information can improve hotspot localization. Nevertheless, deep segmentation models generally depend on annotated training data and computational resources, while their performance can be influenced by dataset size, imaging conditions, and domain variation [13].

Alongside learning-based approaches, threshold-based segmentation remains an attractive alternative for thermal hotspot analysis because of its simplicity, interpretability, and low computational cost. In such approaches, grayscale thermal images are converted into binary representations through threshold selection. In particular, Otsu’s method automatically determines a global threshold by maximizing inter-class variance and has been applied to thermal inspection tasks [14]. Afifah et al. applied Otsu-based segmentation to PV thermal images and reported an average hotspot detection accuracy of 92.16%, indicating that classical thresholding can provide competitive results for suitably preprocessed thermal data [15]. Accordingly, Otsu-based segmentation provides a relevant conventional baseline for evaluating the additional panel-aware and local-background processing stages introduced in the present framework.

Image enhancement is also important in PV thermography because limited contrast, noise, and non-uniform thermal distributions can reduce the separability of hotspot regions. Conventional techniques such as Histogram Equalization (HE) and Adaptive Histogram Equalization (AHE) improve image contrast but may amplify noise or produce excessive enhancement in relatively homogeneous regions [16]. Contrast-Limited Adaptive Histogram Equalization (CLAHE) addresses this limitation by enhancing local contrast while restricting excessive histogram amplification. CLAHE and related enhancement strategies have therefore been used to improve the visibility of low-contrast structures in noisy images [17]. Although some enhancement methods have been evaluated initially in imaging domains other than PV thermography, their ability to improve local contrast provides a practical motivation for their use as preprocessing operations in thermal hotspot analysis.

Although deep learning-based segmentation and detection approaches are widely used for PV thermal inspection, their practical implementation often requires substantial computational resources and manually annotated datasets. In particular, supervised models such as YOLO require hotspot regions to be labeled while distinguishing them from panel edges, shadows, reflections, and external hot objects, making dataset preparation time-consuming and potentially prone to annotation uncertainty [18]. From a physical perspective, thermographic studies have shown that defective PV regions may exhibit measurable temperature asymmetries and localized thermal variations [19], while UAV-based infrared (IR) inspections have demonstrated their effectiveness in identifying abnormal thermal patterns in large-scale PV systems [20]. However, an elevated local thermal intensity does not necessarily indicate an actual PV fault, as similar patterns may also arise from temporary shading, soiling, reflections, environmental conditions, viewing geometry, scene structure, or sensor artifacts. Therefore, the regions identified by the proposed framework are conservatively interpreted as image-based thermal-anomaly candidates rather than confirmed electrical or material faults. Since electrical measurements and maintenance records were not available for region-by-region verification, no fault-level physical validation is claimed in this study.

### 1.2. Methodological Contribution and Novelty

Gaussian filtering, Otsu thresholding, top-hat filtering, morphological erosion, connected-component labeling, and local background comparison are well-established techniques. Therefore, the contribution of this work does not lie in introducing these operations individually but rather in their systematic integration into an interpretable and computationally lightweight panel-aware framework for PV thermal-anomaly candidate detection and bounding-box pre-annotation. Unlike supervised segmentation approaches that typically require manually annotated training data, the proposed framework is designed to operate directly on thermal images without a dedicated training stage. The primary differentiating component of the proposed framework is the integration of panel-aware spatial constraints with local-background intensity comparison and panel-coverage filtering in Method 3. Rather than evaluating candidate regions solely according to global image intensity, this strategy evaluates thermal-anomaly candidates within the spatial context of the detected PV-panel region and their immediate local surroundings. The main methodological and practical contributions of this study are summarized as follows:

- A row-wise thermal averaging and one-dimensional (1D) Otsu-based panel masking strategy is integrated to constrain the analysis to relevant PV panel regions and reduce background interference.
- Morphological erosion is incorporated to generate a conservative inner-panel mask, reducing boundary-related and off-panel detections.
- Method 3 integrates local-background intensity comparison with panel-coverage filtering to retain spatially consistent thermal-anomaly candidates and suppress irrelevant detections. This combined panel-aware local filtering strategy constitutes the main methodological differentiator of the proposed framework.
- In the absence of pixel-level ground-truth annotations, the methods are comparatively evaluated using detected-region count, panel-coverage ratio, out-of-region ratio, and other defined quantitative indicators.
- The framework is evaluated on both public PV thermal images and real-world UAV thermal images acquired from a grid-connected rooftop PV system at Zonguldak Bülent Ecevit University (BEUN) to examine its behavior under different imaging conditions.

Accordingly, the contribution of this study is primarily practical and methodological, arising from the engineering integration of established image-processing operations into a panel-aware, training-free thermal-anomaly detection and automated pre-annotation pipeline, rather than from novelty in the individual processing components.

We organized this paper as follows: Material and Methods are explained in Section 2. The dataset is also introduced, and the comparative methodologies for PV thermal-anomaly candidate detection are described in this section. The Results and Discussion Section, which is called Section 3, presents both visual and quantitative findings. The paper concludes with Section 4, titled Conclusions. Finally, future work and the limitations of the study are discussed.

## 2. Materials and Methods

### 2.1. Publicly Available Thermal PV Dataset

The experimental evaluation is performed in two stages. In the first stage, the publicly available photovoltaic (PV) thermography dataset provided by Marcos Gabriel on Kaggle [21] is used to develop and validate the proposed thermal-anomaly candidate detection framework. This dataset consists of infrared (IR) thermal images collected from real-world PV installations under outdoor operating conditions. All images represent large-scale PV fields and include variations in environmental illumination, camera angle, atmospheric conditions, and panel aging.

**Figure 1:**
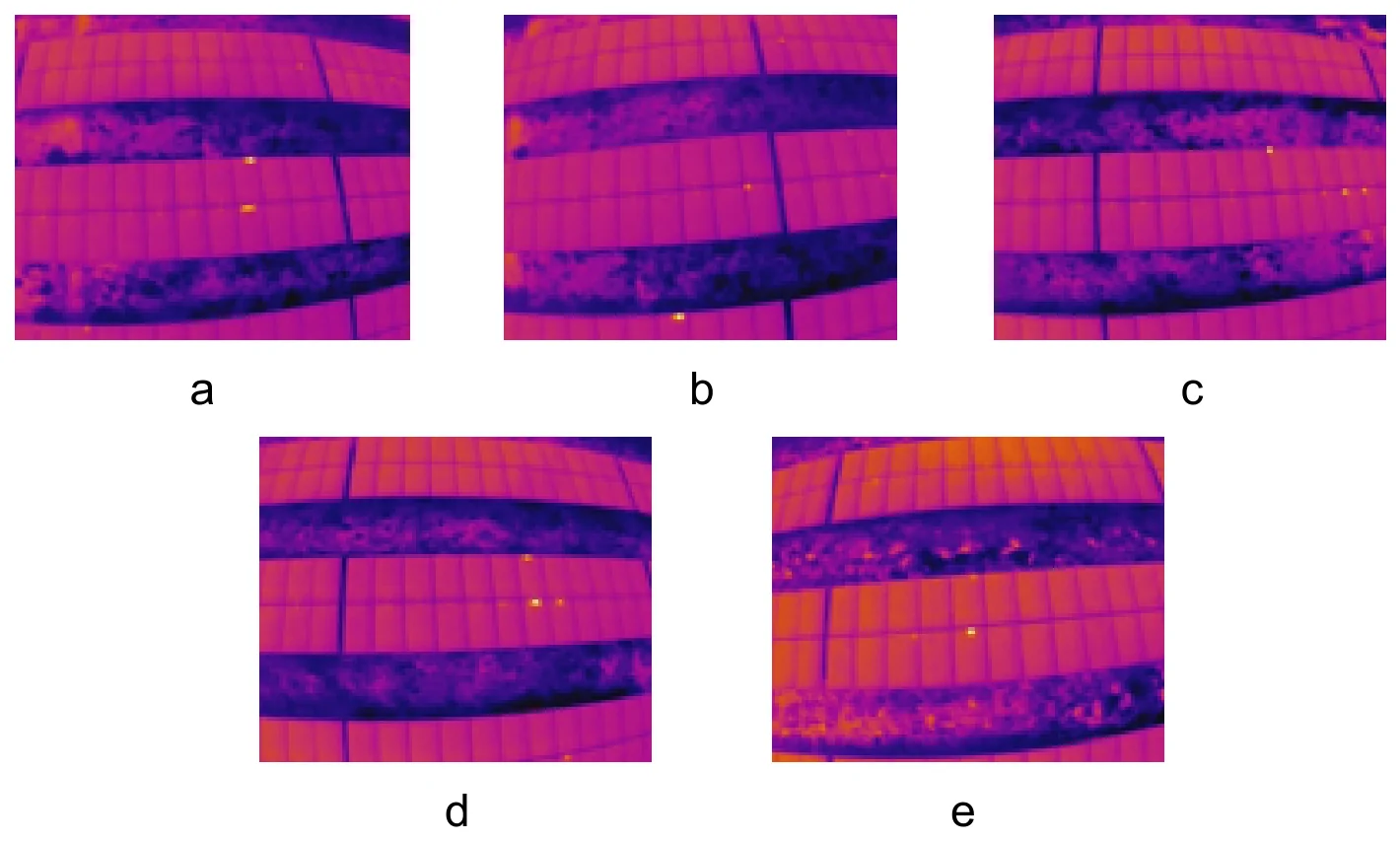
Sample thermal images obtained from a publicly available dataset: (**a**) Image 1, (**b**) Image 2, (**c**) Image 3, (**d**) Image 4, (**e**) Image 5.

The images are acquired under outdoor conditions using an unmanned aerial vehicle (UAV)-based thermal inspection setup. All images are grayscale infrared (IR) thermographic images, captured by a long-wave infrared (LWIR) camera operating typically in the 8–14 µm spectral range, and stored in JPEG format. The spatial resolution of the images varies across different subsets but is approximately 640 × 480 pixels. To ensure the relevance and consistency of the experimental analysis, only images containing clearly visible PV modules are selected from the dataset and used in the thermal-anomaly candidate detection experiments. In this study, a subset of five representative images given in Figure 1 are also selected from the dataset for detailed qualitative and quantitative evaluation.

The images presented in Figure 1 are obtained from a publicly available PV thermal imaging dataset. These images are selected to represent diverse imaging and thermal conditions that may be encountered under real-world PV inspection scenarios. The selected images encompass variations in PV module orientation and viewing perspective, inter-panel spacing, partial panel visibility within the image frame, environmental and structural complexities such as vegetation and supporting structures, and thermal anomalies of varying intensity levels.

In Image 1, the PV array is captured from an oblique viewing angle with pronounced perspective distortion, while part of the array extends beyond the image boundaries. Dark horizontal bands and spatially non-uniform thermal patterns further increase the visual complexity of the scene. Image 2 exhibits partial panel visibility, extensive low-intensity regions, and pronounced variations in thermal intensity, resulting in a spatially heterogeneous background. In Image 3, the PV array is more centrally positioned and captured from a relatively less oblique viewing angle, although only a portion of the array remains within the field of view. The heterogeneous thermal intensity distribution, particularly in the lower region of the image, introduces additional background complexity. Image 4 is characterized by a pronounced oblique viewing geometry and partial panel visibility, together with localized thermal anomalies of moderate to high intensity. Finally, Image 5 contains a prominent thermal anomaly accompanied by a smaller and comparatively lower-intensity region within a complex thermal background. Taken together, the selected images cover a range of geometric and thermal conditions, from variations in viewing geometry to weak and prominent hotspot signatures under heterogeneous background conditions.

This combination provides substantial visual and thermal diversity, enabling the evaluation of thermal-anomaly candidate detection methods not only in terms of their ability to identify prominent high-intensity regions but also in terms of their robustness and reliability against variations in panel orientation, partial panel visibility, heterogeneous thermal patterns, and environmental complexity under realistic PV inspection conditions. Such conditions are particularly challenging for segmentation-based detection approaches, as variations in panel geometry and background characteristics may directly affect the accuracy of panel localization and subsequent thermal-anomaly candidate identification.

The dataset reflects field-acquired PV thermal imaging conditions and includes several sources of visual and thermal complexity. The images exhibit non-uniform background intensity distributions associated with heterogeneous surroundings, such as soil, vegetation, and structural elements. Local intensity variations and high-intensity regions are also observed within or near the PV arrays, some of which may not necessarily correspond to actual PV thermal anomalies and may therefore contribute to false-positive detections. Based on these characteristics, the publicly available dataset was selected to provide diverse and challenging conditions for evaluating the proposed panel-aware thermal-anomaly candidate detection and annotation framework while supporting reproducibility. The variability in viewing geometry, panel visibility, thermal patterns, and background complexity enables the assessment of the framework’s ability to localize relevant PV panel regions, suppress background-related and off-panel detections, and retain spatially consistent thermal-anomaly candidates under varying imaging conditions. Thus, the dataset provides an appropriate basis for evaluating the effectiveness and robustness of the proposed panel masking and filtering strategies.

Despite these advantages, the dataset has several limitations that should be considered in the analysis. It does not provide temperature calibration information or pixel-level/bounding-box ground-truth annotations, preventing direct estimation of absolute temperature values and conventional annotation-based performance evaluation. In addition, variations in UAV viewing geometry, partial panel visibility, and image quality may affect panel localization and thermal-anomaly candidate detection. These limitations were considered in the design and evaluation of the proposed framework, which incorporates panel-coverage filtering, an inner-panel constraint, and local-background comparison to reduce off-panel and background-related false detections.

### 2.2. Real-World Thermal PV Dataset

In the second stage, the applicability of the proposed framework is further assessed using thermal images acquired from a grid-connected rooftop photovoltaic power plant. This second stage evaluation enables the verification of the method under real operating conditions, including challenging scenarios involving solar reflections, panel-edge effects, and out-of-panel thermal artifacts commonly encountered in field-acquired thermal imagery.

The field data used in this study are acquired from a grid-connected rooftop photovoltaic power plant installed on the cafeteria building at the Farabi Campus of Zonguldak Bülent Ecevit University (BEUN). The plant has a total installed capacity of 39.6 kWp and consists of 72 SCHMID (SPE 550-144M) (SCHMID Pekintaş, Düzce, Türkiye) photovoltaic modules rated at 550 Wp each and three HUAWEI SUN2000-15KTL-M5 inverters (HUAWEI, Shenzhen, China). To investigate the effects of panel orientation and surface conditions, the power plant was divided into three independent 13.2 kWp subsystems. Two arrays comprising 48 modules were oriented toward the southwest with a tilt angle of $30^{\circ }$ and an azimuth angle of $15^{\circ }$. The remaining array, consisting of 24 modules, was oriented toward the northeast with a tilt angle of $30^{\circ }$ and an azimuth angle of $-165^{\circ }$.

Thermal inspections were carried out in June 2026 using the integrated thermal camera of a DJI Matrice 30T UAV (DJI, Shenzhen, China) [22]. The thermal camera employs an uncooled VOx microbolometer with a 9.1 mm focal-length lens. Thermal images were acquired in infrared super-resolution mode at 1280 × 1024 pixels. The main technical specifications of the UAV platform and its thermal imaging system are summarized in Table 1.

**Figure 2:**
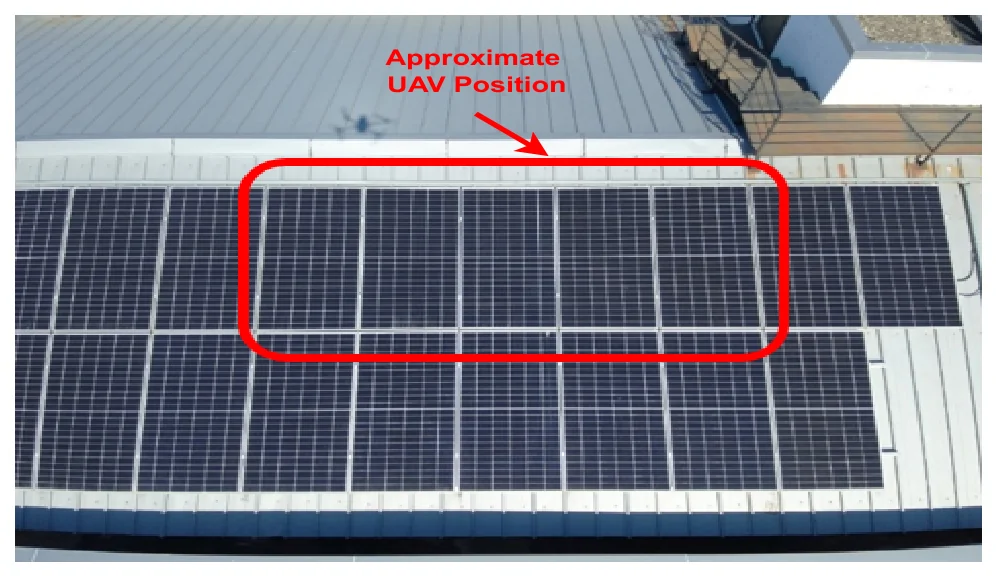
Aerial view of the rooftop PV system and UAV thermal inspection area and approximate UAV position during thermal image acquisition.

For the purposes of this study, the displayed minimum and maximum temperature limits were predefined, and a purple-yellow color palette was selected for thermal visualization during image acquisition. The resulting field images were stored as pseudo-color thermal images with the corresponding camera-displayed temperature scale retained in each image; for the images evaluated in this study, the displayed scale was approximately 20 °C to 50 °C. However, the corresponding raw radiometric data required to obtain calibrated pixel-wise temperature values were not available for the present analysis. Therefore, the displayed temperature scale is used only to provide thermal context for visual interpretation and is not used for quantitative temperature estimation or temperature-based fault diagnosis. The proposed image-processing framework instead operates on the visual thermal representation and relative image-intensity patterns, with the field evaluation focusing on the spatial localization of thermal anomalies. Figure 2 presents an aerial overview of the rooftop PV plant and provides the spatial context of the thermal inspection, including the inspected PV arrays, the corresponding thermal inspection area, and the approximate UAV position during image acquisition.

**Figure 3:**
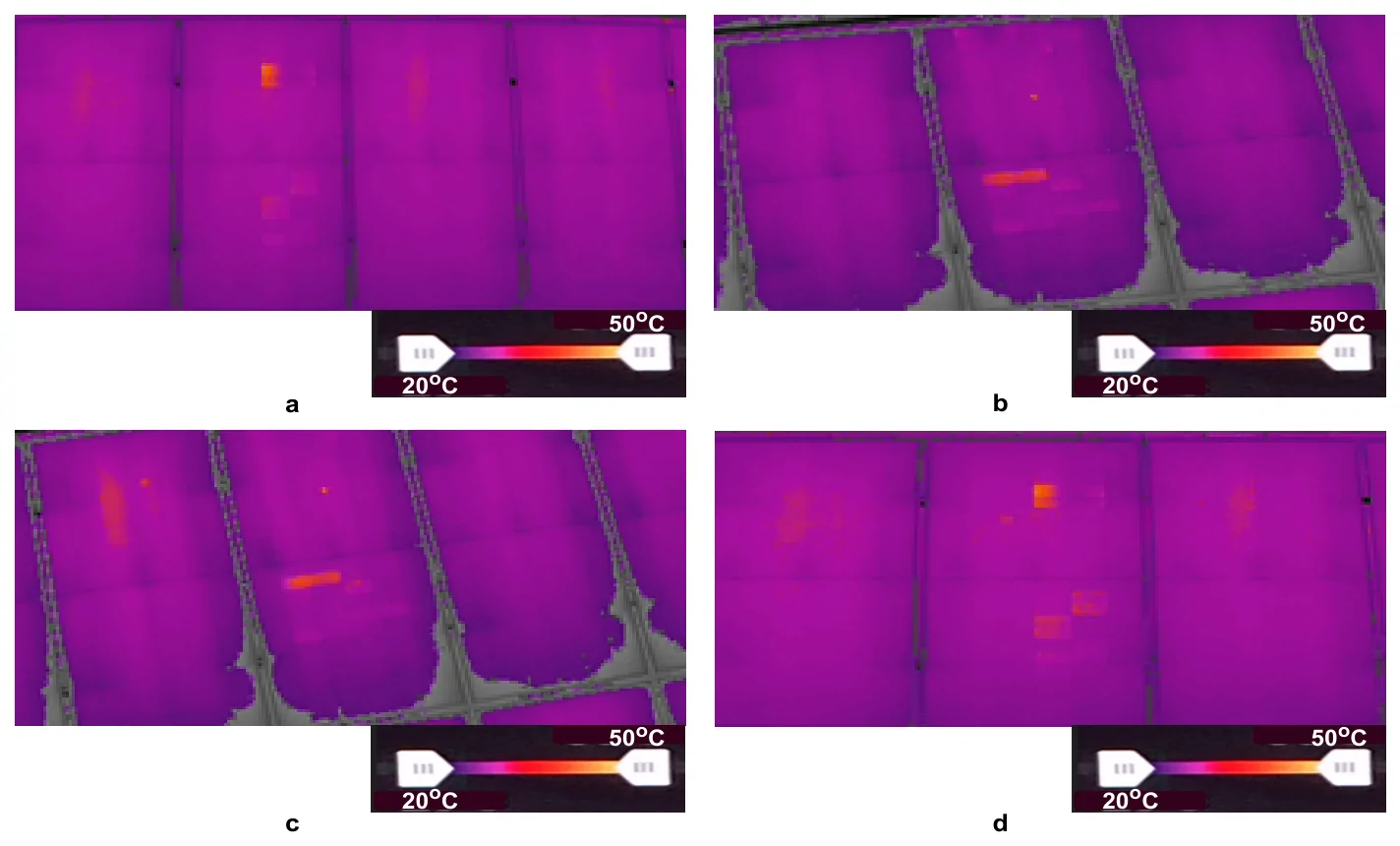
Representative pseudo-color thermal images acquired during UAV-based PV inspections with image-specific temperature scales: (**a**) Image 1, (**b**) Image 2, (**c**) Image 3, (**d**) Image 4.

To evaluate the robustness of the proposed framework beyond controlled benchmark datasets, real-world field experiments are conducted using a UAV equipped with a high-resolution thermal imaging system. During the inspections, the UAV hovered above the operating solar power plant and acquired thermal imagery under varying daylight and environmental conditions. Four representative thermal images acquired during UAV-based inspections of the photovoltaic power plant are selected and are presented in Figure 3.

Figure 3 presents four representative pseudo-color thermal images acquired during UAV-based inspections of the PV power plant under different viewing geometries and field conditions. During image acquisition, the color palette and the displayed minimum and maximum temperature limits were manually defined, and the corresponding temperature scale was retained in each image. However, raw radiometric data providing calibrated pixel-wise temperature values were not available for the present analysis; therefore, the proposed method operates on relative thermal-intensity patterns rather than calibrated pixel-wise temperature data. The images contain multiple PV modules, panel frames, inter-module gaps, and surrounding regions with varying thermal characteristics, providing challenging real-world conditions for evaluating the proposed framework.

### 2.3. Methodology

**Figure 4:**
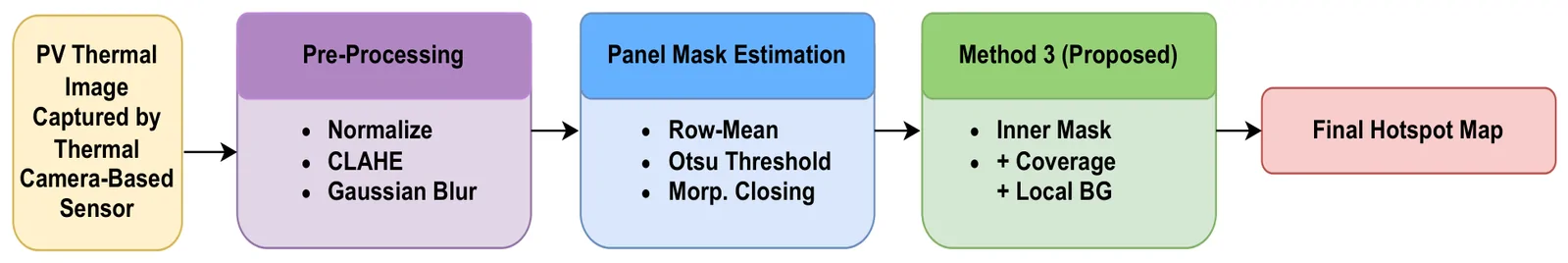
Schematic overview of the proposed methodological framework.

In this section, the methodologies employed for the development and evaluation of three distinct thermal-anomaly candidate detection methods using thermal images of photovoltaic (PV) modules are presented. Figure 4 presents a schematic overview of the proposed methodological framework, illustrating the integrated processing pipeline for solar photovoltaic (PV) panel analysis and thermal-anomaly candidate detection. This proposed methodological framework includes the sequential stages involved in panel localization and thermal anomaly recognition.

The first stage (Method 1) uses a global thresholding strategy without relying on panel segmentation. The second (Method 2) incorporates a panel mask to eliminate non-panel regions. Finally, the proposed approach (Method 3) introduces additional structural and statistical constraints to improve robustness against false detections while preserving valid thermal-anomaly candidates.

#### 2.3.1. Method 1: (Global) Contrast-Based Thermal-Anomaly Candidate Detection

This method is a fundamental preprocessing approach for detecting bright anomalies in thermal images. First, the thermal image is converted to grayscale and normalized to the 8-bit range. Contrast-Limited Adaptive Histogram Equalization (CLAHE) is then applied to enhance local contrast and make low-contrast thermal-anomaly candidate regions more distinguishable. Unlike conventional Adaptive Histogram Equalization (AHE), CLAHE divides the image into small contextual regions (tiles) and performs local histogram equalization while limiting excessive contrast amplification through a predefined clip limit. The clipped histogram values are redistributed among the gray levels, and neighboring tiles are combined using bilinear interpolation to reduce boundary artifacts. Consequently, CLAHE improves the visibility of local thermal anomalies while suppressing excessive noise amplification, providing a more suitable input for the subsequent thermal-anomaly candidate detection stage [17,23].

Morphological top-hot transformation is performed to separate small and bright structures from the background. In this context, the mathematical framework used in this study is based on classical image processing techniques and statistical decision theory. In the conventional top-hat approach, target regions are extracted by subtracting the morphologically opened version of the image from the original image. The opening operation, which consists of an erosion followed by a dilation using a predefined structuring element, is employed to suppress bright objects in images. By carefully selecting the structure element, bright target-like pixels are effectively removed during the opening process, and their difference with the original image highlights these regions [24]. Morphological top-hot transformation is implemented based on the mathematical morphology theory defined by Serra [25]. Local density statistics and sliding window averages are calculated in accordance with standard methods in the field of computer vision [26]. In the resulting top-hat image, thermal-anomaly candidate regions are extracted by determining a global threshold value representing the top percentile of all pixels (e.g., 99.7%).

Global thresholding is a fundamental image binarization technique in which a single threshold is determined from the gray-level histogram of an entire image or image set to separate foreground and background regions. This approach is particularly effective when the histogram exhibits a bimodal distribution; however, its performance degrades in the presence of noise, low contrast, or overlapping foreground–background intensity distributions. Among global thresholding techniques, Otsu’s method is a widely used, fully automatic, nonparametric, and unsupervised algorithm that determines an optimal threshold by maximizing the variance between-class of gray-level intensities. By modeling the normalized histogram as a probability distribution over L discrete gray levels, the method partitions pixels into two intensity-based classes using a single threshold, allowing efficient object–background separation without requiring prior knowledge, training data, or manual parameter tuning. Due to its simplicity, low computational cost, and ease of implementation, Otsu’s method has been extensively applied in image segmentation tasks [27,28].

In this work, a preprocessing step is performed in the first stage of the proposed model. In this preprocessing stage, all thermal images are transformed into 8-bit grayscale format to achieve uniform intensity scaling throughout the dataset. Local contrast is subsequently enhanced using CLAHE, which enables the effective amplification of temperature gradients associated with thermal-anomaly patterns while maintaining structural integrity. Following contrast enhancement, a morphological top-hat transformation was applied to suppress background components and accentuate locally high-intensity locally bright regions. Thus the identification of candidate thermal anomalies are facilitated.

**Figure 5:**
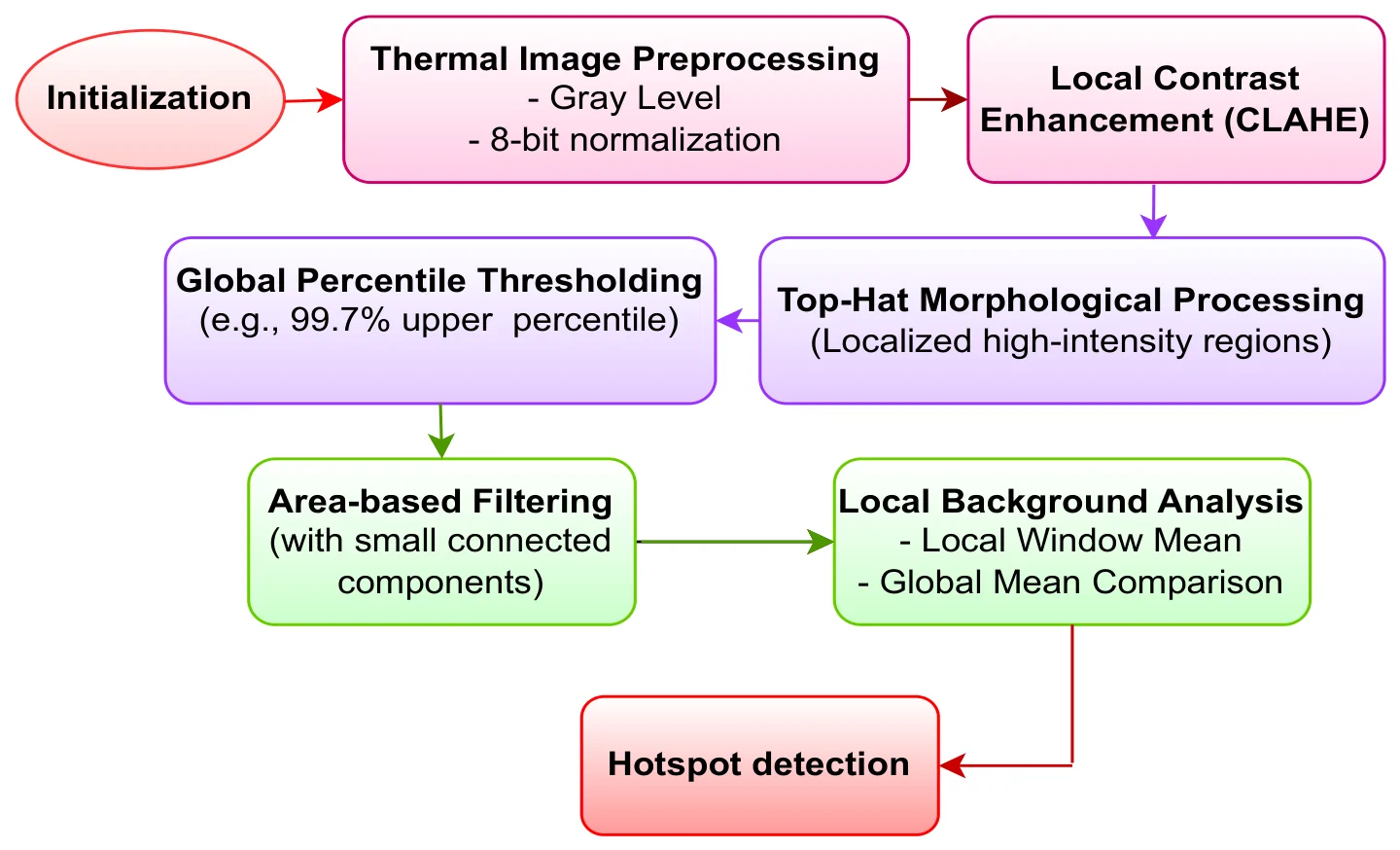
Schematic flowchart of Method 1.

The global statistical thresholding mechanism is inspired by variance-based decision approaches based on Gaussian distribution [29]. In the panel segmentation stage, the Otsu thresholding method, which maximizes the variance between classes, is used [30]. The extraction of regional features (area and centroid) is carried out through via component analysis [31]. The spatial coverage constraint is based on regional overlap principles, which are widely used in object positioning literature. The thermal contrast between the hotspot and the background was quantitatively expressed using local density difference measures. Then, to reduce noise impact, small components below a certain area threshold were eliminated. In the final stage, a local window is defined for each candidate region, and the average of the environmental background is calculated and compared with the global average. Regions that do not sufficiently separate from the background are excluded from the system without being retained as thermal-anomaly candidates. The steps in this method are presented as a flowchart given in Figure 5.

The method shown schematically in Figure 5 does not use a panel mask. Therefore, hot objects outside the panel can also be detected as thermal-anomaly candidates. This increases the number of false positives. For this reason, Method-1 has been improved by adding a mask that limits the panel region and has been named Method-2 in this study.

#### 2.3.2. Method 2: (Baseline) Panel-Based Hotspot Detection

**Figure 6:**
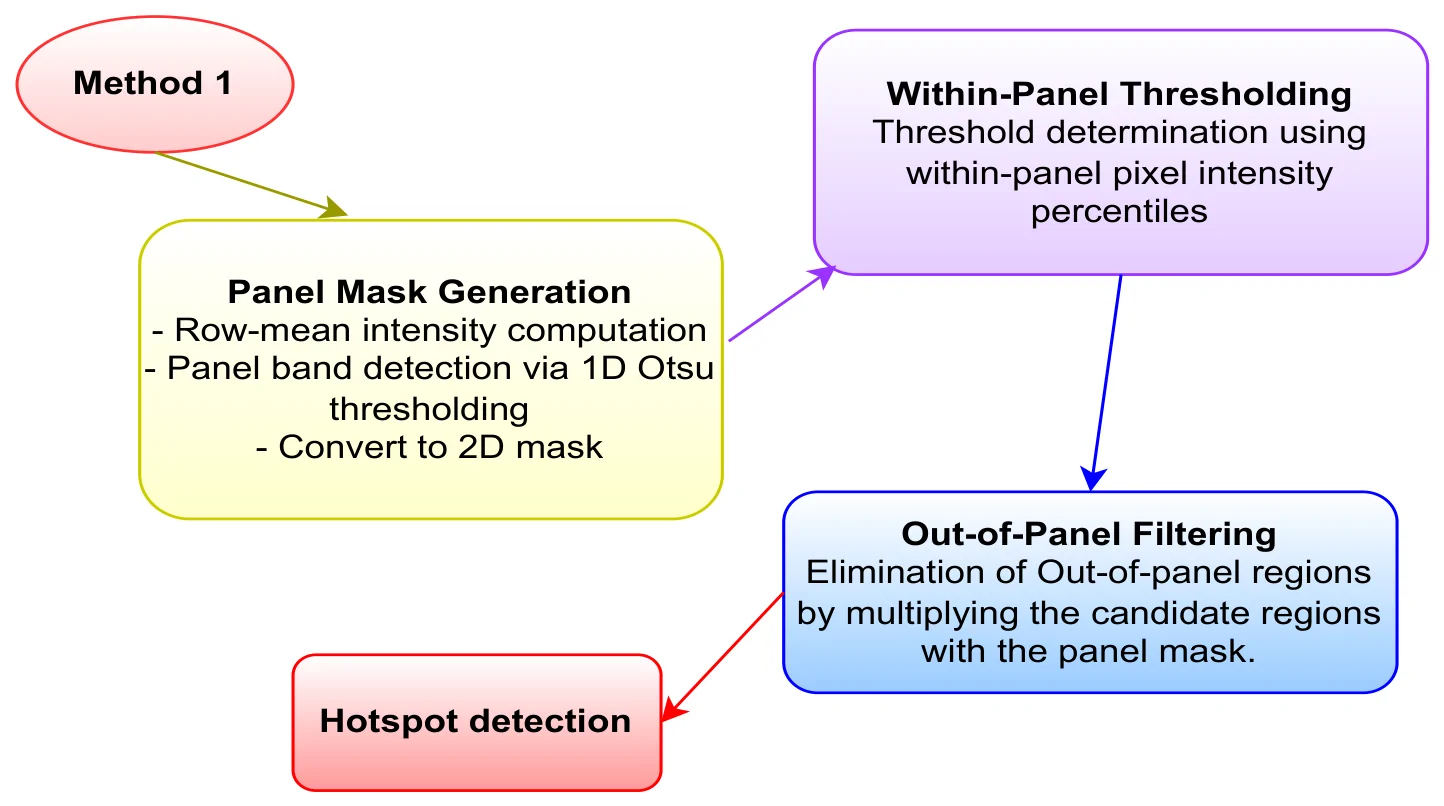
Schematic flowchart of Method 2.

Method 2 is an improved version of Method-1, preserving its basic processing steps while incorporating an additional masking stage that spatially constrains the analysis domain exclusively to the panel region. First, the row-based average gray level values of the image are calculated to automatically determine the panel positions. Panel bands are identified by applying the Otsu thresholding method to the resulting 1-dimensional density profile. These identified bands are expanded to the image size to create a two-dimensional panel mask. Subsequently, rather than performing thresholding on a global image basis, the operation is carried out using the pixel percentage density distribution computed exclusively over the panel mask, thereby restricting the decision process to panel-internal intensity statistics. This prevents the background outside the panel from affecting the threshold value and allows for more precise detection of potential anomalies within the panel. In the final stage, the candidate regions obtained after thresholding are subjected to a mask multiplication with the predefined panel mask, thereby completely eliminating all regions outside the panel boundaries and strictly confining the analysis to the physically significant panel area. This approach contributes to reducing false positives and increasing the accuracy and reliability of panel-based anomaly detection. The sequential processing steps of Method-2, formally designated as the (Baseline) Panel-Based Hotspot Detection, are systematically illustrated in the flowchart presented in Figure 6.

The masking strategy used in Method 2, which limits the panel region, significantly reduces false positive detections originating outside the panel. Moreover, high-density pixels arising from thermal noise or edge effects in regions proximal to the panel boundaries may still be preserved as thermal-anomaly candidates under the current thresholding approach. Although this method calculates the local background average (*local_mean*), this statistical measure is used only for quantitative reporting of detected regions and comparative analyzes. It is not directly involved in the decision-making or filtering process. The local mean can be mathematically formulated as presented in Equation (1).

(1) $$\text{local\_mean}=\frac{1}{M\;N}\sum_{x=1}^{M}\sum_{y=1}^{N}I\left(x,y\right)$$

Here, M,N and I(x,y) denote the total number of pixels and the pixel intensity value in the image, respectively. This method does not provide additional discrimination that would suppress spatial context or local contrast differences. However, it exhibits limited selectivity, particularly in complex regions near panel edges.

#### 2.3.3. Method 3: (Proposed) Advanced Local Background-Based Hot Spot Detection

Method 3 retains the panel-based masking approach of Method 2 but is enhanced with additional spatial and statistical constraints to enhance the spatial accuracy of detected thermal-anomaly candidates and more effectively suppress background effects. First, to eliminate alignment errors, reflection-induced brightness, and boundary ambiguities that might occur at panel edges, the existing panel mask is subjected to morphological erosion. Thus, an inner panel mask is obtained that represents only reliable inner panel regions. For each detected candidate region, a localized background filter centered at the hotspot centroid is analyzed. The candidate region is rejected if the following criterion is not satisfied:

(2) local_mean>μ+kσ

where μ, σ denote the global image mean and global standard deviation, respectively. k<0 represents a negative bias constant introduced to adjust the decision threshold. Following these processes, the overlap ratio between the defined bounding box and the panel area is calculated for each candidate hotspot. A requirement is introduced that this ratio must be above a certain threshold (e.g., 40%) to eliminate weakly correlated areas. Additionally, by making it mandatory for the geometric center of the hotspot to be located within the inner panel mask boundaries, the aim is to systematically filter out ambiguous detections near the panel edge or extending beyond the panel. The ratio of panel pixels inside the bounding box was computed by using Equation (3).

(3) $$\mathrm{coverage}=\frac{A_{\text{panel/in}}}{A_{\mathrm{bbox}}}$$

Here, $A_{\text{panel/in}}$ denotes the number of panel pixels contained within the bounding box (BBox), whereas $A_{\mathrm{bbox}}$ represents the total number of pixels within the bounding box. Candidates with coverage <0.4 are rejected by eliminating off-panel reflections, metallic structures, and environmental artifacts.

(4) $$\text{Out of Region (Panel) Ratio}=\frac{\text{Number of off-panel hotspots}}{\mathrm{Total}\;\mathrm{number}\;\mathrm{of}\;\mathrm{hotspots}\;(K)}$$

**Figure 7:**
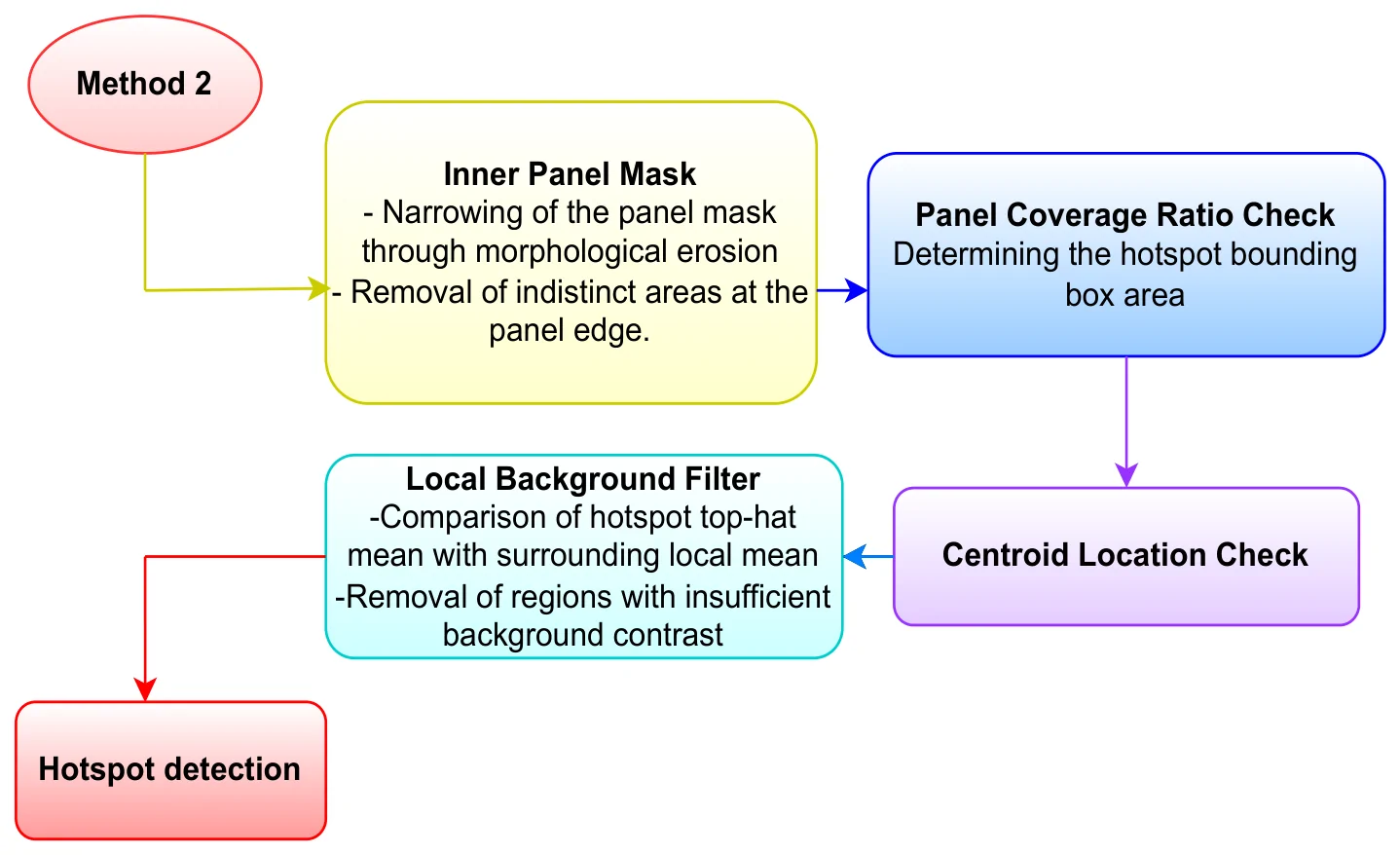
Schematic flowchart of Method 3.

In the final stage of this method, the average top-hat response of each hotspot region is compared to the average value of a defined local window around it. Regions that do not show sufficient statistical separation from the background are then eliminated. Thanks to this multi-stage filtering strategy, the method both reduces the false positive rate and allows for more reliable differentiation of true thermal anomalies within the panel. The sequential processing steps of this method are illustrated in the flowchart presented in Figure 7.

Method 3 effectively suppresses false positive detections originating outside the panel area and errors due to boundary effects, reflections, and geometric ambiguities occurring at panel edges. Thanks to the applied internal panel masking, coverage ratio, and center position constraints, it is made mandatory for the detected candidate regions to have a strong spatial relationship with the panel surface. In addition, a filtering stage based on local background comparison is incorporated into the detection pipeline. This stage suppresses candidate regions exhibiting insufficient thermal contrast relative to their surrounding neighborhood. Furthermore, regions that do not demonstrate statistically significant deviation from the local background temperature distribution are discarded. Consequently, only thermally distinct and spatially localized temperature elevations occurring on the panel surface are retained and classified as valid hotspots.

For each accepted hotspot, two main quantitative metrics are calculated. Firstly, the mean top-hat intensity (brightness), which is called *comp_val*, is calculated by using Equation (5).

(5) $$\text{comp\_val}=\frac{1}{N}\sum_{i=1}^{N}T_{i}$$

Here, $T_{i}$ and *N* denotes the top-hat response (output) pixel intensity and Component pixel count, respectively. Moreover, the relative intensity difference represents the thermal contrast between the hotspot region and its local background. It serves as a key indicator for assessing and comparing hotspot prominence across methods. Local intensity contrast metric is represented as $\Delta I_{\mathrm{mean}}$ and it is given in Equations (6)–(8).

(6) $${\bar{I}}_{H}=\frac{1}{N_{H}}\sum_{p\in H}I\left(p\right)$$

(7) $${\bar{I}}_{B}=\frac{1}{N_{B}}\sum_{p\in B}I\left(p\right)$$

(8) $$\Delta I_{\mathrm{mean}}={\bar{I}}_{H}-{\bar{I}}_{B}$$

where *H* denotes the retained candidate region, *B* denotes the surrounding local-background region, and I(p) is the 8-bit grayscale intensity. Pixels belonging to the candidate region are excluded from B to prevent the candidate itself from influencing the background estimate.

Accordingly, a positive $\Delta I_{\mathrm{mean}}$ indicates that the retained region is brighter than its immediate local background. $\Delta I_{\mathrm{mean}}$ is used as a local contrast descriptor of retained detections rather than as a standalone measure of detection accuracy. It does not represent an absolute temperature difference (°C).

#### 2.3.4. Parameter Selection and Configuration

The parameters used in the proposed framework were fixed before the final evaluation and applied consistently to all evaluated images without image- or dataset-specific adjustment. The selected values were determined through preliminary experiments to provide a practical balance between local contrast enhancement, suppression of background variations, spatial filtering, and computational stability. In particular, the 31 × 31 erosion kernel defines the conservative inner-panel region by removing boundary pixels from the generated panel mask. These values should therefore be regarded as fixed design parameters of the present proof-of-concept implementation rather than globally optimized settings. The complete parameter configuration used in the experiments is summarized in Table 2.

A systematic quantitative sensitivity analysis was not performed in the present study. Therefore, the influence of key parameters on the number of detected regions, out-of-region ratio, and $\Delta I_{\mathrm{mean}}$ will be systematically investigated using a larger and more diverse dataset in future work.

## 3. Results and Discussion

### 3.1. Evaluation of Hotspot Detection Performance on Publicly Available Thermal Image Datasets

In this study, thermal-anomaly candidate locations on photovoltaic panels are identified using three distinct approaches. Method 1 ((Global) Contrast-Based Hotspot Detection) offers low computational complexity. However, it is highly sensitive to external noise and non-panel thermal artifacts. Method 2 ((Baseline) Panel-Based Hotspot Detection) suppresses detections originating from outside the panel region by incorporating a panel mask strategy, yet it still suffers from a relatively high false-positive rate. The proposed Method 3 (Advanced Local Background-Based Hotspot Detection) integrates multiple complementary constraints and validation steps in a unified framework. In this method, inner-panel mask, panel-coverage rule, and local background filter are used together. Method 3 aims to minimize spurious thermal-anomaly candidate detections while maximizing detection reliability and robustness. These performance levels cannot be consistently achieved using the first two methods alone. Within the scope of this study, to more clearly emphasize the performance of the obtained results, Table 3 summarizes the operations utilized and the processing steps applied for three methods.

In addition to Table 3, the filtering operations employed in this study are presented in Table 4.

**Figure 8:**
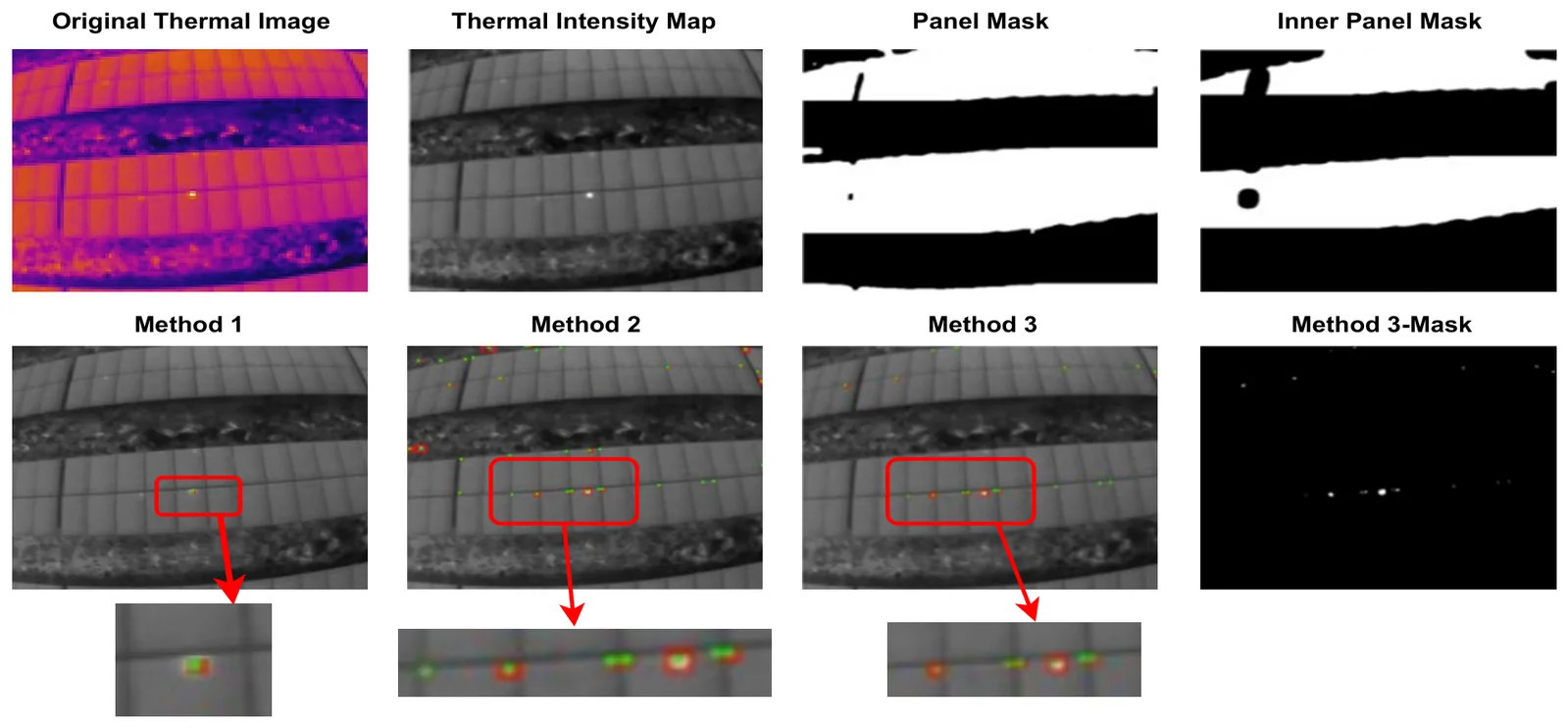
Results of the three methods on the publicly available dataset.

Using all the processing steps, filtering operations, and their functions given above, hotspots are extracted in the PV thermal images given in Figure 1. The results presented in Figure 8 correspond to the three defined methods.

As shown in Figure 8, in the first method, only the local background filter is applied to detect hotspot regions. However, accurate hotspot locations could not be reliably identified using this approach. In the second method, in addition to the first method, row-mean and Otsu Thresholding are incorporated. Although this approach resulted in a higher number of detected hotspot candidates, detections also occurred outside the panel region. Consequently, false detections are produced beyond the actual hotspot locations of interest. In the proposed third method, an additional masking operation is introduced. Thanks to this panel-constrained masking step, only hotspot regions located strictly on the panel surface are detected.

**Figure 9:**
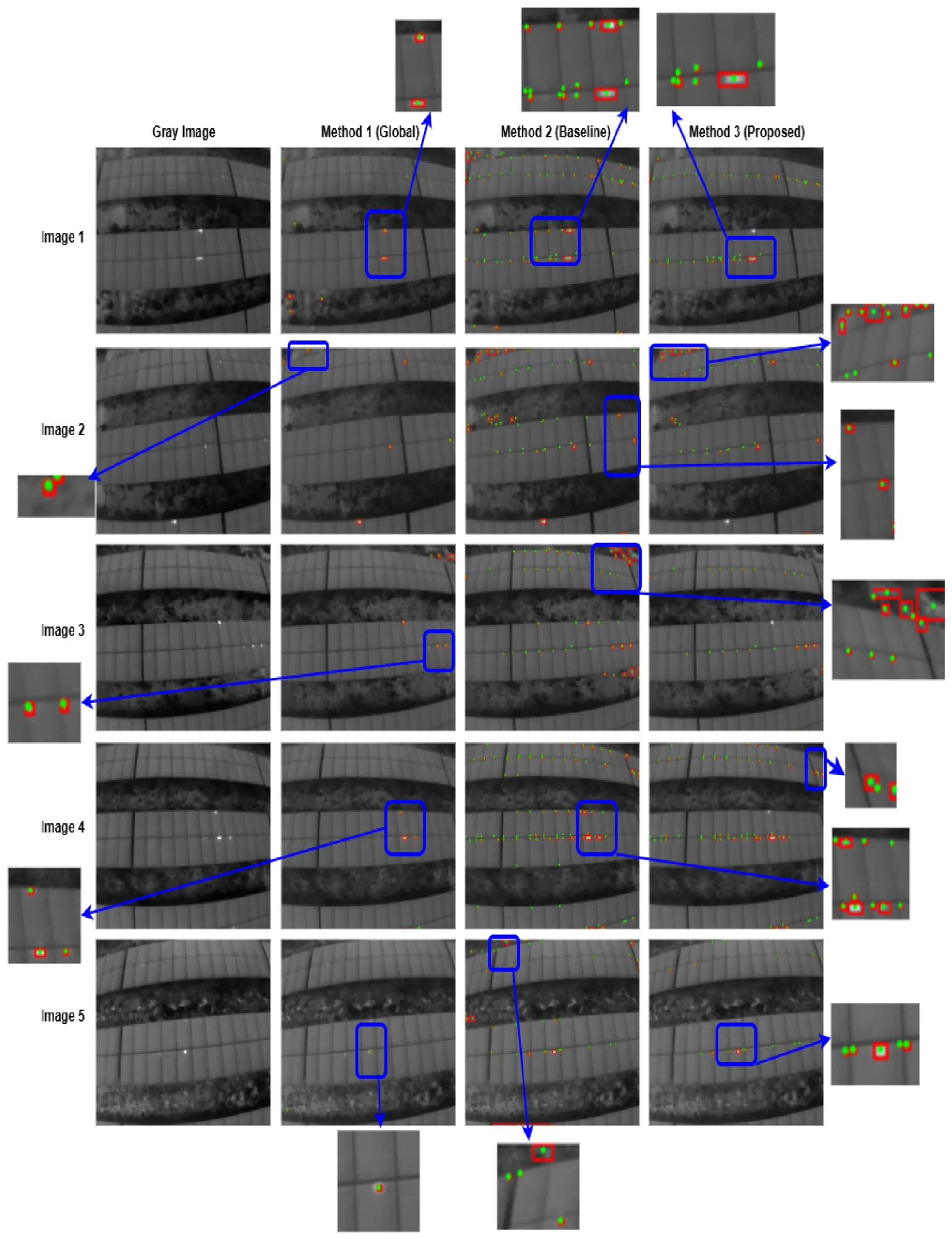
Hotspot detection results for the three methods on the public thermal-image subset.

Based on Figure 8, the hotspot detection results obtained from the three methods for five different images are visually presented in Figure 9. While Figure 8 illustrates the processing steps for the first example image, Figure 9 provides a clearer visual comparison by presenting only the final hotspot detection results for the three methods across multiple sample images.

As illustrated in Figure 9, the Method 1 erroneously classifies high-intensity regions outside the panel boundaries, such as ground surfaces, sky reflections, and metallic glare, as hotspots due to the absence of spatial constraints. Although the Method 2 incorporates a panel mask, it continues to detect high-intensity artifacts near panel edges because it lacks sufficient mechanisms to suppress edge-related noise and residual non-panel effects within the masked region. In contrast, the proposed method (Method 3) exclusively identifies localized hotspots confined to the panel cells and completely eliminates off-panel regions from detection. This outcome demonstrates the effectiveness of the geometry-aware inner-mask refinement combined with local background consistency testing in achieving physically coherent and spatially constrained hotspot detection.

Figure 9 presents the hotspot detection results obtained using the three methods on the public thermal-image subset, including the detected hotspot regions. Furthermore, the off-panel ratio (Out-of-Region Ratio) metrics, mean intensity difference ($\Delta I_{\mathrm{mean}}$), average area, and calculation time of detected hotspot regions are also provided. All results are quantitatively presented in Table 5, separately for five different visualizations and each of the three methods.

As shown in Table 5, Method 1 produces relatively few detections and generally high $\Delta I_{\mathrm{mean}}$ values (37.08–91.07). However, its out-of-region ratio reaches 0.67 in some images, indicating limited spatial selectivity. Method 2 restricts the analysis more effectively to the panel regions but generates substantially more detected regions (37–85), suggesting a tendency to retain numerous local intensity variations. In comparison, Method 3 reduces the number of detected regions relative to Method 2 in all five images (18–58 regions) and generally provides lower out-of-region ratios (0.00–0.34), including a zero out-of-region ratio for Images 4 and 5. These results indicate that the proposed combination of the inner panel mask, panel-coverage criterion, and local-background filtering provides a more spatially constrained set of thermal-anomaly candidates.

$\Delta I_{\mathrm{mean}}$ values quantify the grayscale intensity elevation of each retained candidate relative to its immediate local background. Accordingly, positive $\Delta I_{\mathrm{mean}}$ values indicate that the retained region is locally brighter than its surrounding background. Method 1 exhibits the highest $\Delta I_{\mathrm{mean}}$ values because it retains a small number of high-intensity regions, some of which may occur outside the relevant panel area. Method 2 and Method 3 produce lower $\Delta I_{\mathrm{mean}}$ values, reflecting the retention of more moderate local intensity variations within the analyzed regions. Importantly, $\Delta I_{\mathrm{mean}}$ is not interpreted as an independent measure of detection accuracy; a high value does not necessarily indicate a more reliable thermal anomaly. Therefore, $\Delta I_{\mathrm{mean}}$ is evaluated together with the number, size, and spatial distribution of the detected regions and the out-of-region ratio when comparing the three methods.

To comprehensively evaluate the spatial localization behavior of the three methods, thermal-anomaly candidate detection metrics are computed for five representative test images, enabling a comparative assessment of the detected thermal-anomaly distributions. These distributions are presented in Table 6. In addition to the total number of detected thermal-anomaly candidate regions, their spatial agreement with a pseudo-reference is evaluated to assess localization consistency. The detected regions are categorized as reference-matched candidates (R-MC), partially matched candidates (P-MC), reference-unmatched candidates (R-UC), and out-of-region detections. The developed procedure is tested by all authors, and the resulting pseudo-reference masks are jointly reviewed and visually verified. During this verification, the masks are examined to ensure that the retained regions were located within the PV-panel area and that obvious panel-frame, inter-panel gap, and out-of-panel artifacts were excluded. These categories describe spatial agreement only and do not represent independently verified physical PV fault conditions. Accordingly, the visual-verification step represents a joint author assessment rather than an independently established physical ground-truth procedure.

Since physically validated hotspot annotations based on calibrated temperature measurements, thermocouple measurements, or field inspection records were unavailable for the analyzed thermal images, a pseudo-reference was established solely for image-level spatial comparison. The pseudo-reference regions were derived from the thermal images using a predefined procedure independent of the evaluated detection methods (M1–M3). Reference hotspot regions were generated using local statistical thresholding within the PV-panel regions, followed by morphological cleaning and visual verification to remove obvious panel-frame and out-of-panel artifacts. The resulting binary reference masks were encoded with pixel values of 0 for background and 255 for reference regions. These values are categorical mask labels and do not represent absolute temperature or thermal intensity. In this work, the pseudo-reference has not been interpreted as fault-level ground truth.

The detections obtained by methods (M1–M3) were subsequently categorized according to their spatial relationship with the pseudo-reference regions. A detected hotspot region fully enclosed by a corresponding pseudo-reference region was classified as a reference-matched candidate, whereas a detection partially enclosed by or overlapping with a pseudo-reference region was classified as a partially matched candidate. A detection located within the PV-panel region but showing no spatial agreement with any pseudo-reference region was classified as a reference-unmatched candidate, while detections located outside the defined PV-panel region were categorized as out-of-region detections.

As shown in Table 6, the proposed method (Method 3) generally produced fewer detected regions than the baseline method (Method 2) while substantially reducing out-of-region detections. For Images 1–5, the total number of detected regions is reduced from 73 to 42, 59 to 38, 48 to 22, 85 to 58, and 37 to 18, respectively. In particular, out-of-region detections are eliminated in Images 4 and 5 and substantially reduced in Images 1–3. The distributions of reference-matched, partially matched, and reference-unmatched candidates further indicate that the proposed filtering provides a more spatially constrained set of detections relative to the image-derived pseudo-reference.

Across the five public thermal images, the proposed method retained a total of 178 image-level thermal-anomaly candidates, which were subsequently converted into bounding-box pre-annotations. The variation in the number of retained candidates among the images reflects differences in image content and local thermal-intensity patterns. These results demonstrate the capability of the proposed pipeline to transform automatically detected thermal-anomaly regions into structured, YOLO-compatible pre-annotations.

**Figure 10:**
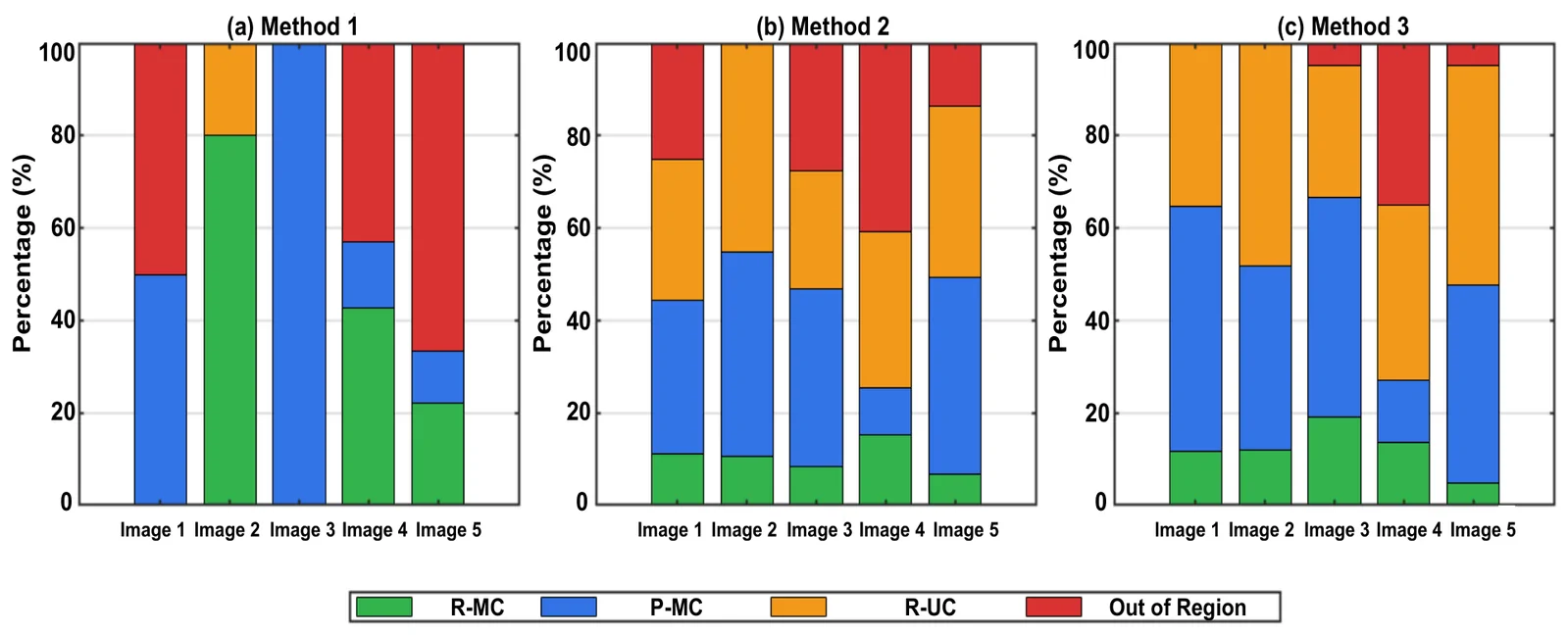
Percentage distribution of hotspot categories for (**a**) Method 1 (Global), (**b**) Method 2 (Baseline), and (**c**) Method 3 (Proposed).

Figure 10 presents the percentage distribution of hotspot candidate categories generated by the proposed method across five representative test images. Each bar represents 100% of the detected hotspot candidates and is partitioned into four categories: reference-matched candidates, partially matched candidates, reference-unmatched candidates, and out-of-region candidates. This representation illustrates the relative proportion of each candidate category for the corresponding test image and provides a comparative assessment of the spatial agreement between the detected hotspot candidates and the reference regions.

The image-wise analysis demonstrates that the proposed method consistently suppresses redundant and out-of-region hotspot candidates across all test images while producing a cleaner and more interpretable hotspot distribution. The detected hotspot candidates were categorized as reference-matched, partially matched, or reference-unmatched according to their spatial correspondence with the reference regions. The proposed method effectively reduced the overall number of candidates and restricted a larger proportion of retained detections to the panel regions. In contrast, Method 1, which does not employ panel masking, generated a considerable number of out-of-region candidates, particularly along image boundaries and outside the panel regions. Although Method 2 incorporates panel masking, its threshold-based hotspot extraction without additional geometric constraints resulted in excessive candidate detections, including partially matched and reference-unmatched candidates associated with residual edge-related and non-panel artifacts. The proposed method, through its additional spatial and geometric filtering criteria, more effectively suppresses these irrelevant candidates.

While the proposed method retains fewer reference-matched candidates in some cases, particularly for Images 1, 2, 4, and 5, the retained detections are more spatially concentrated within the panel regions. This indicates that the proposed filtering strategy favors spatially constrained candidates while suppressing out-of-region and weakly overlapping candidates. Consequently, the proposed method prioritizes the spatial reliability and interpretability of hotspot localization rather than simply maximizing the number of detected candidates, demonstrating robustness against background noise, edge-induced intensity artifacts, and environmental variability. The Out-of-Region Ratio values obtained for the evaluated methods were 0–67% for Method 1, 0–41% for Method 2, and 0–34% for Method 3. These results further indicate that the proposed method substantially suppresses out-of-region candidates and achieves performance comparable to or better than Method 2, depending on the evaluated image.

On the other hand, the image-processing pipeline is implemented in Python (3.10.11) using OpenCV (4.8.1) and NumPy (1.24.3). All computational experiments were performed on a laptop equipped with an Intel(R) Core(TM) i7-8550U CPU operating at 1.80 GHz, 16 GB RAM, and an NVIDIA GeForce MX150 GPU with 2 GB dedicated memory. Although a discrete GPU with CUDA 11.1 support was available, GPU acceleration was not used, and all image-processing operations were executed on the CPU using standard OpenCV/NumPy functions. The average processing times were 19.8–47.3 ms for Method 1, 57.6–145.0 ms for Method 2, and 65.8–152.2 ms for Method 3. The higher computational cost of Method 3 results from the additional processing stages, including inner-mask generation, panel-coverage analysis, and local-background verification. The reported times correspond only to the execution of the detection algorithm and exclude image loading, figure generation/visualization, and annotation-file export. Therefore, these values represent the computational cost of the detection stage rather than the complete end-to-end processing time. The observed processing times indicate that the proposed method is computationally lightweight and shows potential for near-real-time applications. However, end-to-end real-time deployment is not evaluated in the present study.

### 3.2. Evaluation of Photovoltaic Panel Surface Hotspot Detection Performance Using Solar Power Plant Data

**Figure 11:**
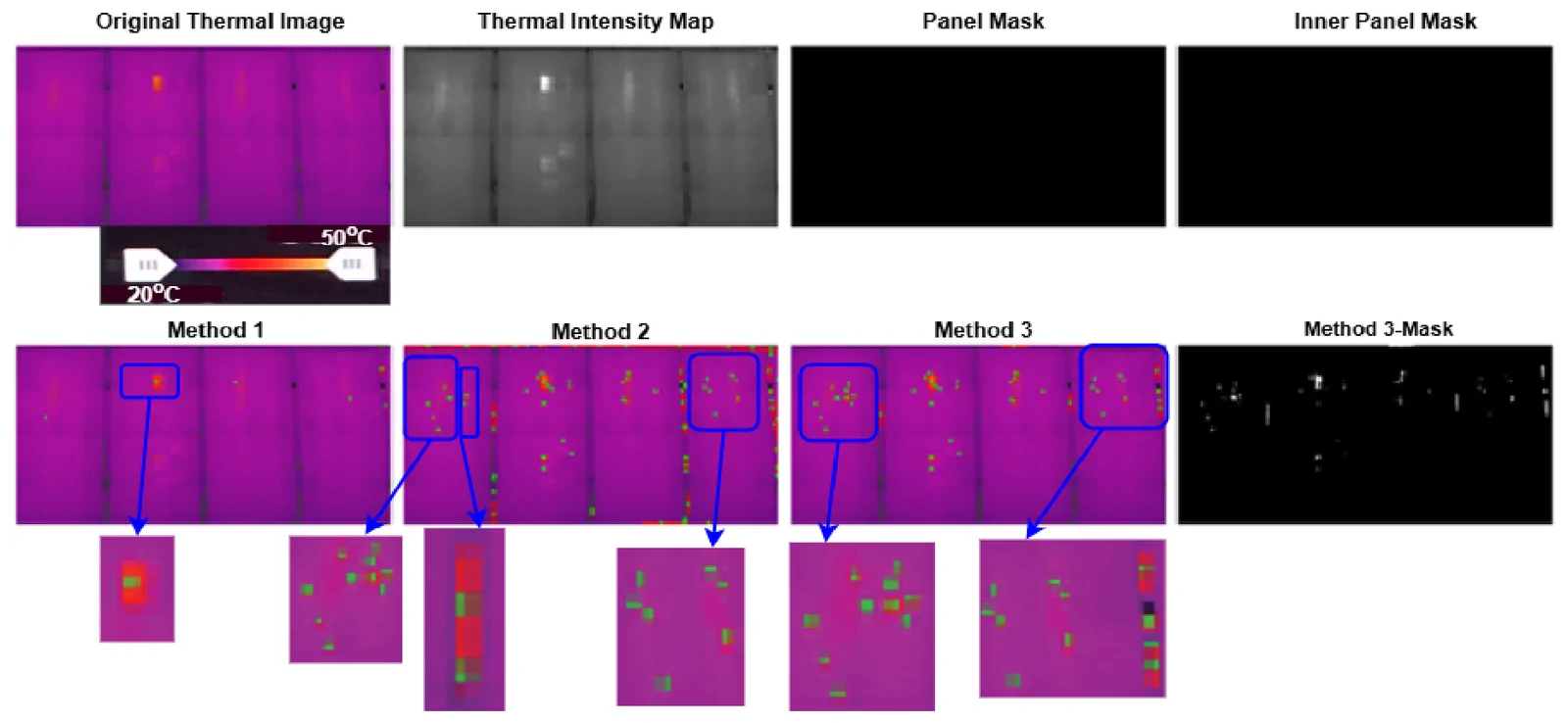
Results for Image 1 Using Real UAV Thermal Data from BEUN Rooftop PV Plant.

**Figure 12:**
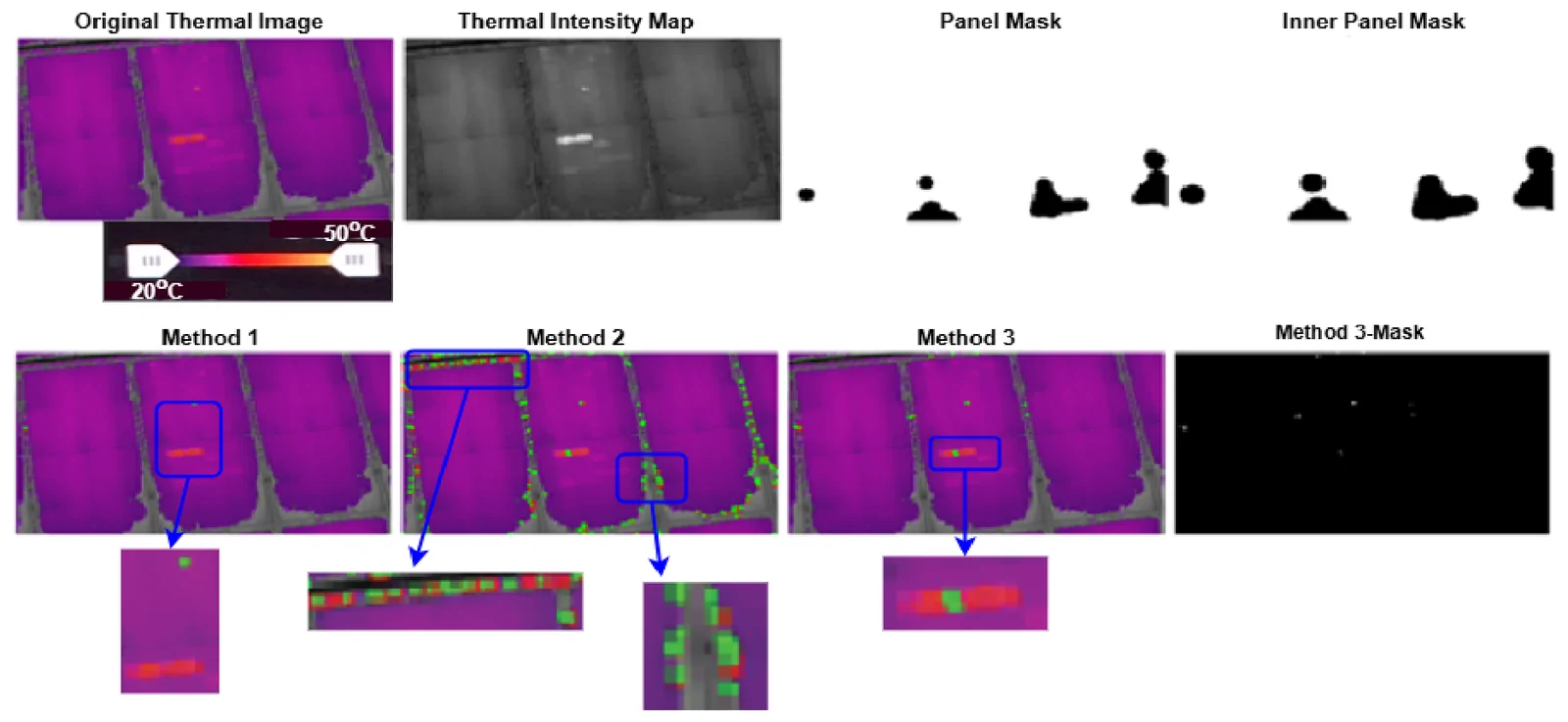
Results for Image 2 Using Real UAV Thermal Data from BEUN Rooftop PV Plant.

**Figure 13:**
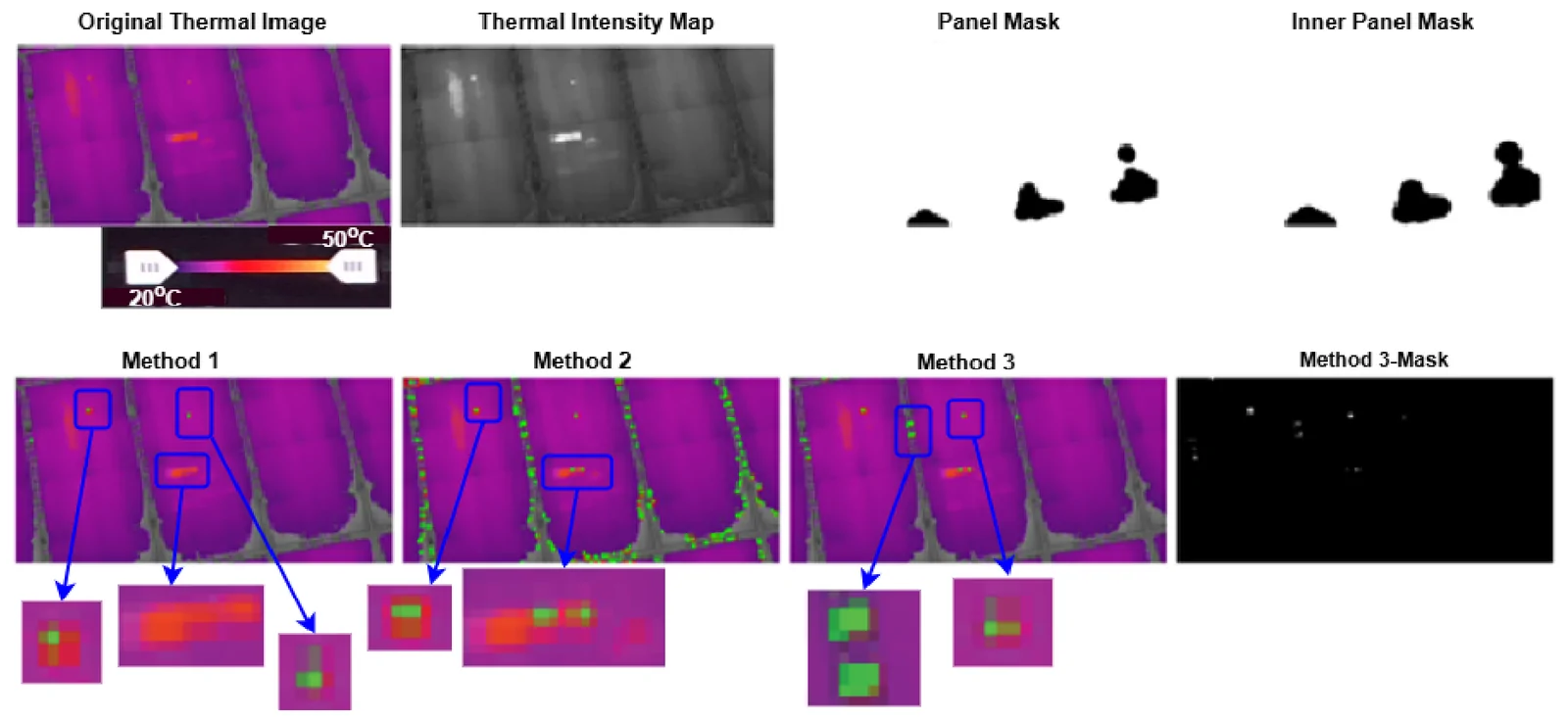
Results for Image 3 Using Real UAV Thermal Data from BEUN Rooftop PV Plant.

**Figure 14:**
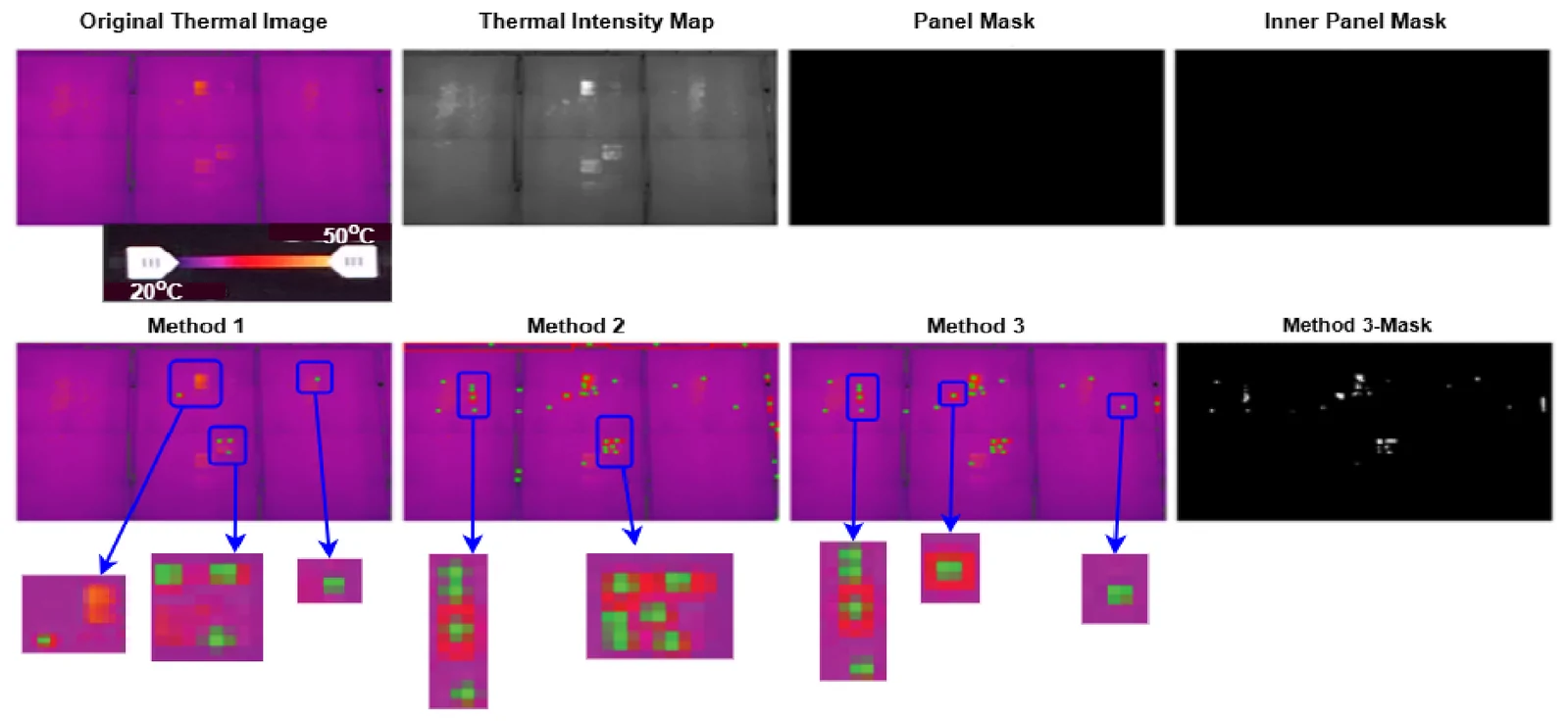
Results for Image 4 Using Real UAV Thermal Data from BEUN Rooftop PV Plant.

The primary objective of this study is to develop a preprocessing and annotation framework capable of automatically generating consistent and reliable hotspot labels for future deep learning-based hotspot detection and classification applications. To this end, the proposed framework is evaluated in two stages. First, its performance is assessed using publicly available photovoltaic thermal datasets acquired under benchmark conditions. Subsequently, its applicability under real-world operating conditions is validated using UAV-based thermal images acquired from a grid-connected rooftop photovoltaic power plant located at the Farabi Campus of Zonguldak Bülent Ecevit University (BEUN). The real-world thermal images, shown in Figure 3, are processed according to the methodology described in Section 3.1 by applying the complete sequence of preprocessing steps, filtering operations, and associated functions. This evaluation enables the assessment of the proposed framework under challenging field conditions, including solar reflections, panel-edge effects, and out-of-panel thermal artifacts. For each retained thermal-anomaly candidate, the proposed method automatically determines the corresponding bounding-box coordinates, providing structured pre-annotation information that can be converted into standard annotation formats used by object-detection frameworks such as YOLO and Faster R-CNN. The thermal-anomaly localization results obtained using the three methods, together with representative examples of the automatically generated bounding-box pre-annotations, are presented in Figure 11, Figure 12, Figure 13 and Figure 14. The real-world field images are acquired using the integrated thermal camera of a DJI UAV and stored as pseudo-color thermal images. Within the scope of this study, the color palette and the displayed minimum and maximum temperature limits are defined during image acquisition, and the corresponding temperature scale is retained in the images. However, raw radiometric data providing calibrated pixel-wise temperature values are not available for the present analysis. Therefore, the proposed method operates on visual thermal representations and relative thermal-intensity patterns rather than calibrated temperature fields. The field evaluation focuses on spatial thermal-anomaly localization rather than quantitative temperature estimation or temperature-based fault diagnosis.

The quantitative results corresponding to the hotspot visualizations presented in Figure 11, Figure 12, Figure 13 and Figure 14 are summarized in Table 7 for each of the three methods and the four representative test images.

Table 7 presents a quantitative comparison of the three thermal-anomaly candidate detection methods in terms of detected-region count, mean intensity difference ($\Delta I_{\mathrm{mean}}$), out-of-region ratio, average detected-region area, and computational time. The proposed method produces substantially fewer detected regions than the baseline while maintaining a considerably smaller average detected-region area. Compared with the baseline, it also achieves a lower out-of-region ratio for Images 1 and 4, indicating improved spatial localization in these images. The larger ($\Delta I_{\mathrm{mean}}$), values obtained by the proposed method for Images 2, 3, and 4 indicate that the retained candidate regions exhibit stronger local grayscale contrast relative to their immediate backgrounds. These values represent solely local contrast descriptors and do not independently indicate higher detection accuracy or better localization performance. Accordingly, the interpretation of ($\Delta I_{\mathrm{mean}}$), together with the detected-region count, out-of-region ratio, and average detected-region area, provides complementary information for characterizing the detection results. Although the computational time of the proposed method is higher than that of the global method, it remains lower than that of the baseline for all test images.

To further evaluate the spatial distribution and correspondence of the detected thermal-anomaly candidates, Table 8 presents the comparison of hotspot evaluation metrics obtained from the rooftop solar power plant images.

As shown in Table 8, the proposed method (Method 3) substantially reduces the total number of detected thermal-anomaly candidates compared with the baseline method (Method 2) for all four rooftop PV images. The number of detected regions decreases from 80 to 42, 150 to 12, 153 to 15, and 41 to 28 for Images 1–4, respectively. Method 3 also reduces the number of out-of-region detections from 42 to 7 in Image 1, from 69 to 9 in Image 2, from 90 to 11 in Image 3, and from 13 to 2 in Image 4. In addition, the proposed method retains the same number of reference-matched candidates as Method 2 for Images 1–3 while retaining 7 compared with 8 for Image 4. These results indicate that the proposed filtering strategy substantially suppresses excessive and out-of-region detections while preserving most of the reference-matched candidates under real-world rooftop PV conditions.

Compared with the results obtained from the public thermal-image subset in Table 6, the real-world rooftop images present more challenging conditions, as reflected by the higher proportion of out-of-region detections for the proposed method, particularly in Images 2 and 3. In the public dataset, Method 3 produced 18–58 detected regions with out-of-region ratios ranging from 0.00 to 0.34, whereas in the real-world dataset it produced 12–42 detected regions with out-of-region ratios ranging from 0.07 to 0.75. Notably, for Images 2 and 3, the out-of-region ratios obtained with Method 3 (0.75 and 0.73, respectively) were higher than those obtained with Method 2 (0.46 and 0.58, respectively), indicating reduced spatial selectivity of the proposed method for these field images. Nevertheless, Method 3 consistently reduced the total number of detected regions relative to Method 2 and substantially reduced the absolute number of out-of-region detections. These findings indicate that the proposed method maintains its candidate-suppression capability under real-world operating conditions, although its relative spatial selectivity is affected by the increased variability and complexity of field-acquired thermal images.

One factor that may contribute to this behavior is the quality of the automatically generated panel mask. Potential mask failure cases can occur when the thermal-intensity contrast between the PV panels and the surrounding background is weak or spatially nonuniform. In particular, strong reflections, thermally similar background regions, panel-edge effects, perspective distortion, and partially visible panels may cause the panel mask to extend into adjacent background regions or incompletely represent the actual panel area. Since the subsequent candidate-filtering stages depend on the generated panel mask, such mask-localization errors can propagate to the final thermal-anomaly localization results. The conservative inner-panel mask employed in Method 3 helps reduce boundary-related leakage; however, it cannot completely eliminate the influence of panel-mask inaccuracies under challenging field conditions.

**Figure 15:**
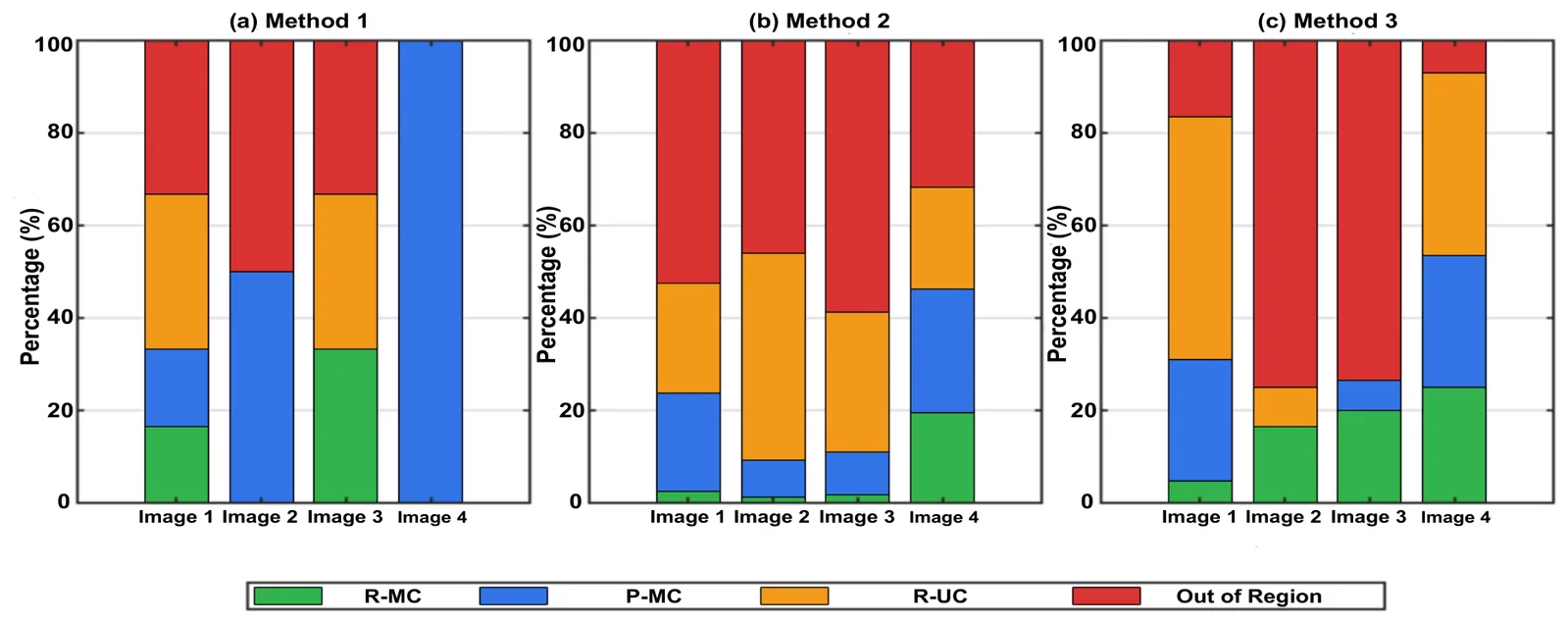
Percentage distribution of hotspot for real thermal images for (**a**) Method 1 (Global), (**b**) Method 2 (Baseline), (**c**) Method 3 (Proposed).

Figure 15 illustrates the percentage distribution of hotspot categories generated by the proposed method using real thermal images acquired from the grid-connected rooftop PV power plant.

In addition to all these considerations, the classical image-processing approach was selected to provide a computationally lightweight and training-free framework while reducing the initial manual annotation effort. The computational results support this design choice. For the five public thermal images, Method 3 required 65.8–152.2 ms per image, while processing times of 74.2–158.2 ms per image were obtained for the four real-world UAV images. Thus, the proposed detection pipeline operates within approximately 66–158 ms per image on the specified test hardware, without requiring a dedicated model-training stage. However, this value represents the maximum runtime within the present proof-of-concept dataset rather than a dedicated worst-case benchmark under systematically controlled levels of clutter, shadows, or scene complexity. This computational profile indicates promising efficiency and potential for near-real-time processing, although a direct quantitative comparison with deep learning-based approaches would require evaluation under identical hardware and dataset conditions. In addition, the training-free framework automatically generates candidate bounding-box pre-annotations, thereby reducing the initial manual annotation effort. However, the corresponding reduction in annotation time has not been quantitatively measured in the present study.

## 4. Conclusions

In this study, a lightweight computer vision approach integrating panel structure, local background statistics, and morphological analyses for thermal-anomaly detection and automated pre-annotation in PV thermal images is proposed. The proposed method offers three key contributions. First, it employs row-mean analysis together with a robust Otsu-based panel mask for panel-region extraction. Second, it introduces inner-mask cellular region analysis and panel-coverage criteria to reduce detections associated with panel boundaries. Third, a local background-based validation mechanism is applied to retain locally significant thermal-anomaly candidates within the analyzed panel regions.

The experimental results obtained from both the publicly available thermal PV dataset and the real-world rooftop PV images reveal differences between relatively controlled benchmark data and practical field conditions. On the public thermal-image subset, the proposed method consistently reduced the number of detected regions compared with the baseline, producing 18–58 candidates with out-of-region ratios of 0.00–0.34. Across the five public images, 178 thermal-anomaly candidates were retained and represented by automatically generated bounding boxes that can be converted into standard annotation formats used by object-detection frameworks. In the real-world rooftop PV images, the proposed method also substantially reduced the total number of candidates compared with the baseline, from 80 to 42, 150 to 12, 153 to 15, and 41 to 28 for Images 1-4, respectively. The corresponding out-of-region detections were reduced from 42 to 7, 69 to 9, 90 to 11, and 13 to 2.

This performance variation may be attributed, in part, to a domain shift between the two datasets. The publicly available dataset consists of grayscale thermal images, whereas the field dataset is composed of pseudo-color thermal images in which thermal information is visually represented through a color map. Consequently, converting pseudo-color images into grayscale may alter the underlying intensity distribution used by the hotspot detection algorithm. This difference in image representation, together with more challenging field conditions, including perspective distortions, varying panel orientations, shadows, surrounding objects, solar reflections, panel-edge effects, and non-uniform thermal-intensity patterns, may contribute to the differences in spatial localization performance observed between the two datasets.

Despite the performance variation between the two datasets, the proposed method maintained its candidate-suppression capability and substantially reduced the total number of detected regions and the absolute number of out-of-region detections relative to the baseline. However, the higher out-of-region ratios observed for some field images, particularly Images 2 and 3, indicate reduced relative spatial selectivity under challenging real-world conditions. These findings further highlight the importance of evaluating thermal-anomaly localization algorithms under real-world PV operating conditions rather than relying exclusively on controlled public datasets.

Overall, the results demonstrate that the proposed framework is applicable to both public and field-acquired thermal images and can automatically generate spatially constrained bounding-box pre-annotations for subsequent deep learning applications. The generated bounding-box coordinates can be converted into standard annotation formats required by object-detection frameworks such as YOLO and Faster R-CNN. However, these deep learning models were not trained or evaluated in the present study. Therefore, the generated bounding boxes can be regarded as pre-annotations intended to facilitate subsequent dataset preparation rather than as validated ground-truth annotations. Residual false-positive or imperfectly localized detections may occur, particularly under challenging field conditions. Therefore, manual verification and correction remain necessary before the generated annotations are used for model training. For segmentation-based architectures such as Mask R-CNN, the generated bounding boxes can serve as initial regions of interest, while additional pixel-level annotations are required for segmentation. Thus, the proposed framework primarily aims to reduce the manual effort associated with identifying and localizing candidate thermal anomalies and to provide a structured starting point for future deep learning-based detection and classification studies.

## 5. Limitation and Future Works

A key objective of this study is to establish a preprocessing and automated pre-annotation framework capable of generating consistent thermal-anomaly candidate labels for future deep learning-based detection and classification studies. Nevertheless, several limitations should be considered when interpreting the present results. First, the proof-of-concept evaluation is limited to nine thermal images, comprising five images from a public dataset and four independently acquired real-world rooftop PV images. Therefore, the reported results should not be interpreted as evidence of broad generalization, robustness across different PV systems, or field-deployment readiness. In addition, differences between the grayscale public images and the pseudo-color field-acquired images, together with variations in perspective, panel orientation, reflections, background conditions, and thermal-intensity distributions, may affect the performance of the image-intensity-based detection procedure. Because the proposed method relies on gray-level intensity rather than calibrated radiometric temperature, its output may also be affected by camera-dependent acquisition and image-generation settings, including exposure, gain, dynamic-range scaling, and pseudo-color palette mapping. These factors can modify the gray-level representation and local intensity contrast used in the detection procedure, particularly when pseudo-color images are converted to grayscale. Their individual effects were not systematically quantified in the present proof-of-concept study and will require controlled evaluation using different cameras and acquisition settings in future work. In addition, the principal algorithmic parameters, including the panel percentile, inner-mask erosion size, local-background window, (bg_k) and minimum coverage ratio, were treated as fixed design parameters in the present study, and a systematic quantitative sensitivity analysis was not performed. Therefore, no claim is made regarding the robustness of the proposed framework to variations in these parameters. Future work will systematically vary the principal parameters around their nominal values and quantify their effects on the detected-region count, out-of-region ratio, ($\Delta I_{\mathrm{mean}}$), and related performance measures using larger and more diverse field datasets.

Another limitation concerns the quantitative validation of the automatically generated PV-panel masks, which constitute an intermediate processing stage of the proposed framework. In the present study, representative mask overlays were used for qualitative verification, while a dedicated manually delineated panel-mask reference dataset was not established. Consequently, quantitative segmentation metrics such as Intersection over Union (IoU) or Dice score are not reported. Future work will establish a manually delineated reference subset covering both public and more challenging field-acquired images, enabling quantitative evaluation of panel-mask accuracy using IoU and Dice metrics. This will also allow for the propagation of panel-localization errors to the subsequent thermal-anomaly candidate detection stage to be systematically investigated.

A third limitation concerns the thermal information available for the analysis. The proposed framework operates on gray-level local contrast ($\Delta I_{\mathrm{mean}}$), rather than calibrated pixel-wise temperature values. For the field-acquired images used in this study, raw radiometric data providing calibrated pixel-wise temperature values are not available. Although the displayed temperature scale is retained in the pseudo-color thermal images, it does not provide direct access to calibrated temperature values for individual pixels. Consequently, $\Delta I_{\mathrm{mean}}$, represents the relative gray-level difference between a candidate region and its local background and should not be interpreted as an absolute temperature difference (**ΔT**). The present framework therefore focuses on spatial thermal-anomaly localization rather than quantitative temperature estimation or temperature-based fault diagnosis. In addition, the field images were analyzed after conversion from pseudo-color thermal representations to grayscale, while alternative approaches using the original color-space features directly were not evaluated in the present study. Therefore, the potential information retained in the pseudo-color representation and the influence of different color-space representations on thermal-anomaly localization remain to be investigated. Future work will compare grayscale-based processing with direct color-space analysis and, where available, calibrated radiometric thermal data.

A further limitation is the absence of independently validated physical ground truth for thermal anomalies in all analyzed images. The pseudo-reference used in this study was derived from the thermal images and was employed solely to evaluate the spatial correspondence of the detected candidates. The resulting pseudo-reference masks were jointly reviewed and visually verified by all authors; however, this procedure does not constitute an independent physical ground-truth measurement or a formal inter-observer agreement assessment. Accordingly, reference-matched, partially matched, reference-unmatched, and out-of-region categories represent image-level spatial relationships rather than independently confirmed PV fault conditions. Moreover, the proposed framework does not explicitly model the electrical or heat-transfer mechanisms responsible for thermal-anomaly formation. Since corresponding electrical measurements, maintenance records, calibrated temperature measurements, and independent fault-diagnosis data are not available for all analyzed images, confirmation of the physical origin and severity of the detected thermal anomalies remains beyond the scope of the present study. Future work will therefore include a formally defined multi-evaluator verification protocol and quantitative inter-observer assessment to further improve the reproducibility of the pseudo-reference construction and evaluation procedure.

Future work will address these limitations by evaluating the framework on substantially larger and more diverse datasets covering different PV technologies, weather conditions, seasons, times of day, acquisition geometries, and thermal imaging characteristics. New thermal datasets will be acquired using radiometric thermal imaging systems capable of providing calibrated pixel-wise temperature data. The radiometric analysis will account for camera-specific calibration and relevant measurement parameters, including PV-module surface emissivity, camera-to-panel distance, reflected apparent temperature, ambient temperature, relative humidity, and atmospheric transmission. This will enable the current gray-level local-contrast analysis to be extended toward cell- and region-based **ΔT** evaluation in °C. The detected anomalies will also be validated using complementary information such as electrical measurements, repeated thermographic observations, maintenance records, and on-site inspections where available, enabling a more rigorous assessment of both spatial localization performance and the physical relevance of the detected thermal anomalies.

Finally, the proposed framework will be investigated as an automated pre-annotation tool for larger PV thermal-image datasets. The generated panel-aware candidate regions can be represented as structured bounding-box pre-annotations and converted into standard annotation formats used by object-detection frameworks such as YOLO and Faster R-CNN. For segmentation-based architectures such as Mask R-CNN, the generated bounding boxes can serve as initial regions of interest, while additional pixel-level annotations are required for segmentation. Although no deep learning training or recognition experiments were conducted in the present study, future work will evaluate the quality of the generated pre-annotations through downstream model training and independent test-set performance. Integration of automated pre-annotation, radiometric **ΔT** analysis, and deep learning-based detection represents a future direction toward a scalable PV thermography analysis framework for practical inspection applications.

## Figures and Tables

**Table 1:** Main technical specifications of the UAV platform and thermal imaging system used for field data acquisition.

Parameter	Specification
UAV weight	3770 ± 10 g (including two batteries)
Maximum flight time	41 min
Maximum wind resistance	12 m/s
GNSS	GPS + Galileo + BeiDou
Thermal detector	Uncooled VOx microbolometer
Thermal lens	9.1 mm, f/1.0
Thermal camera DFOV	61°
Thermal image resolution	640 × 512 (normal); 1280 × 1024 (super-resolution)
Pixel pitch	12 μm
NETD	≤50 mK @ F1.0
Temperature measurement accuracy	±2 °C or ±2%, whichever is greater
Temperature measurement range	−20 to 150 °C (High Gain); 0 to 500 °C (Low Gain)

**Table 2:** Parameters used in the implementation of the proposed methods.

Parameter	Value	Role in the Implementation
CLAHE clip limit	2.0	Local contrast enhancement
CLAHE tile grid	8×8	CLAHE local tiling
Top-hat kernel	17×17	Emphasize small localized bright structures
Global percentile (Method 1)	99.7	Global threshold
Panel percentile (Methods 2/3)	98.5	Within-panel adaptive threshold
min_area (Method 1)	8 pixels	Small-component filtering
min_area (Methods 2/3)	3 pixels	Small-component filtering
Local-background window	41×41	Local context
bg_k (Method 1)	−0.1	Local-background criterion
bg_k (Method 3)	−0.5	Local-background criterion
Inner-mask erosion	31×31	Exclude panel-boundary region
Panel coverage minimum	0.40	Minimum bbox/panel overlap constraint
Panel-mask Gaussian blur	31×31	Panel-mask preprocessing
Vertical dilation	6 pixels	Expand row-based panel bands
Closing kernel	7×7	Fill panel-mask gaps
Opening kernel	3×3	Remove small noise components
top_k	None	No fixed candidate-count cap

**Table 3:** Processing steps for three method.

Category	Process/Filter	M1 Global	M2 Baseline	M3 Proposed
Pre-processing	8-bit normalize	+	+	+
	Gaussian Blur (panel mask)	-	+	+
Panel Segmentation	Row-mean calculation	-	+	+
	1D Otsu (row mean)	-	+	+
	2D Otsu	-	+	+
	Dilation (panel mask)	-	+	+
	Closing (hole filling)	-	+	+
Contrast Enhancement	CLAHE	+	+	+
and Hotspot Detection	White Top-Hat (17 × 17)	+	+	+
Thresholding	Global percentile threshold	+	-	-
	Panel-internal percentile threshold	-	+	+
Noise Reduction	Opening (3 × 3)	+	+	+
Connected Component Labeling	Hotspot candidate generation	+	+	+
(CCL)	Area filtering (min_area)	+	+	+
Geometric and Statistical Features	comp_val (mean top-hat intensity)	+	+	+
	center coordinate	+	+	+
	area (pixel area)	+	+	+
	local_mean calculation	+ (filter)	+ (report)	+ (filter)
On-Panel Verification (Key distinctions)	Panel mask utilization	-	+	+
	Inner-panel mask (erosion)	-	-	+
	Erosion (31 × 31)	-	-	+
	Panel coverage rate	-	-	+
	Local background (bg) filter (global_mean+bg_coefficientbg_c×std)	+	-	+

**Table 4:** Filtering operations with related function.

Category	Filter	Function
Contrast Filter	CLAHE	Enhances local contrast and increases hotspot discriminability.
Low-pass Filter	Gaussian Blur	Reduces row mean noise during panel mask extraction.
Morphological Filter	White Top-Hat	Highlights local bright regions.
Morphological Filter	Opening (3 × 3)	Removes single-pixel noise.
Morphological Filter	Closing (7 × 7)	Fills holes in the panel mask.
Morphological Filter	Erosion (31 × 31)	Extracts the inner panel region and suppresses edge-related radiance artifacts.
Thresholding	Otsu Threshold	Segments panel rows and bright pixels.
Statistical Threshold	Percentile Threshold	Selects only the brightest pixels as hotspot candidates.

**Table 5:** Quantitative characteristics of detections obtained on the public thermal-image subset.

Image Name	Method	Detected Regions	Δ $I_{\mathrm{mean}}$	Out-of-Region Ratio	Mean Area (Pixel)	Time (ms)
Image 1	Method 1 (Global)	9	40.49	0.67	20.3	48.67
	Method 2 (Baseline)	73	10.09	0.14	10.6	24.30
	Method 3 (Proposed)	42	6.84	0.05	12.9	19.90
Image 2	Method 1 (Global)	7	37.08	0.43	47.43	44.2
	Method 2 (Baseline)	59	9.01	0.41	32.88	135.2
	Method 3 (Proposed)	38	6.34	0.34	32.37	123.2
Image 3	Method 1 (Global)	7	51.10	0.57	59.14	47.32
	Method 2 (Baseline)	48	14.08	0.28	43.67	107.6
	Method 3 (Proposed)	22	9.75	0.05	22.68	97.9
Image 4	Method 1 (Global)	5	67.15	0.00	48.60	46.2
	Method 2 (Baseline)	85	10.41	0.00	22.21	145.0
	Method 3 (Proposed)	58	6.75	0.00	24.60	152.2
Image 5	Method 1 (Global)	2	91.07	0.5	17.00	19.8
	Method 2 (Baseline)	37	16.67	0.24	58.30	57.6
	Method 3 (Proposed)	18	12.86	0.00	17.22	65.8

**Table 6:** The comparison of hotspot evaluation metrics for the public thermal-image subset.

Image	Method	Total Detected Thermal-Anomaly Candidates	R-MC	P-MC	R-UC	Out-of- Region Detections
Image 1	Method 1 (Global)	9	2	1	0	6
	Method 2 (Baseline)	73	5	31	27	10
	Method 3 (Proposed)	42	2	18	20	2
Image 2	Method 1 (Global)	7	3	1	0	3
	Method 2 (Baseline)	59	9	6	20	24
	Method 3 (Proposed)	38	6	5	14	13
Image 3	Method 1 (Global)	7	0	3	0	4
	Method 2 (Baseline)	48	4	18	13	13
	Method 3 (Proposed)	22	5	10	6	1
Image 4	Method 1 (Global)	5	4	0	1	0
	Method 2 (Baseline)	85	9	37	39	0
	Method 3 (Proposed)	58	7	23	28	0
Image 5	Method 1 (Global)	2	0	1	0	1
	Method 2 (Baseline)	37	4	12	12	9
	Method 3 (Proposed)	18	2	10	6	0

**Table 7:** Quantitative characteristics of detections obtained from the real-world rooftop PV thermal images.

Image Name	Method	Detected Regions	Δ $I_{\mathrm{mean}}$	Out-of-Region Ratio	Mean Area (Pixel)	Time (ms)
Image 1	Method 1 (Global)	6	65.40	0.33	38.67	63.5
	Method 2 (Baseline)	80	10.25	0.53	72.00	144.4
	Method 3 (Proposed)	42	6.75	0.16	39.19	131.0
Image 2	Method 1 (Global)	2	85.37	0.50	14.0	76.1
	Method 2 (Baseline)	150	5.25	0.46	15.43	224.7
	Method 3 (Proposed)	12	35.49	0.75	111.42	158.2
Image 3	Method 1 (Global)	3	72.84	0.33	26.67	75.0
	Method 2 (Baseline)	153	5.03	0.58	13.73	187.2
	Method 3 (Proposed)	15	25.43	0.73	17.40	140.5
Image 4	Method 1 (Global)	5	68.52	0.00	24.80	49.7
	Method 2 (Baseline)	41	6.26	0.31	110.56	83.4
	Method 3 (Proposed)	28	11.63	0.07	36.89	74.2

**Table 8:** The comparison of hotspot evaluation metrics obtained from rooftop solar power plant images.

Image	Method	Total Detected Thermal-Anomaly Candidates	R-MC	P-MC	R-UC	Out-of- Region Detections
Image 1	Method 1 (Global)	6	1	1	2	2
	Method 2 (Baseline)	80	2	17	19	42
	Method 3 (Proposed)	42	2	11	22	7
Image 2	Method 1 (Global)	2	0	1	0	1
	Method 2 (Baseline)	150	2	12	67	69
	Method 3 (Proposed)	12	2	0	1	9
Image 3	Method 1 (Global)	3	1	0	1	1
	Method 2 (Baseline)	153	3	14	46	90
	Method 3 (Proposed)	15	3	1	0	11
Image 4	Method 1 (Global)	5	0	5	0	0
	Method 2 (Baseline)	41	8	11	9	13
	Method 3 (Proposed)	28	7	8	11	2

## Data Availability

The original contributions presented in this study are included in the article. Further inquiries can be directed to the corresponding author.

## Abbreviations

The following abbreviations are used in this manuscript:

PV	Photovoltaic
CPU	Central Processing Unit
CNNs	Convolutional Neural Networks
ROI	Region of Interest
U-Net	U-shaped Convolutional Neural Network
SegNet	Semantic Segmentation Network
R-CNN	Region-Based Convolutional Neural Network
YOLO	You Only Look Once
2TSS	Two-Tier Semantic Segmentation
ResNet	Residual Neural Network
HE	Histogram Equalization
AHE	Adaptive Histogram Equalization
CLAHE	Contrast Limited Adaptive Histogram Equalization
1D	One-Dimensional
IR	Infrared
UAV	Unmanned Aerial Vehicle
LWIR	Long-Wave Infrared
R-MC	Reference-Matched Candidates
P-MC	Partially-Matched Candidates
R-UC	Reference-Unmatched Candidates
IoU	Intersection over Union
